# Warming Trends and Bleaching Stress of the World’s Coral Reefs 1985–2012

**DOI:** 10.1038/srep38402

**Published:** 2016-12-06

**Authors:** Scott F. Heron, Jeffrey A. Maynard, Ruben van Hooidonk, C. Mark Eakin

**Affiliations:** 1 NOAA Coral Reef Watch, NESDIS Center for Satellite Applications and Research, 5830 University Research Ct., E/RA3, College Park, MD 20740, USA; 2 Global Science and Technology, Inc., Greenbelt, MD 20770, USA; 3 Marine Geophysical Laboratory, Physics Department, College of Science, Technology and Engineering, James Cook University, Townsville, Qld 4811, Australia; 4 SymbioSeas and the Marine Applied Research Center, Wilmington NC 28411, USA; 5 CRIOBE – USR 3278, CNRS – EPHE – UPVD, Laboratoire d’Excellence “CORAIL”, 58 Av. Paul Alduy - 66860 Perpignan cedex, France; 6 NOAA Atlantic Oceanographic and Meteorological Laboratory, Ocean Chemistry and Ecosystems Division, 4301 Rickenbacker Causeway, Miami, FL 33149, USA; 7 Cooperative Institute for Marine and Atmospheric Studies, Rosenstiel School of Marine and Atmospheric Science, University of Miami, 4600 Rickenbacker Cswy., Miami, FL 33149, USA

## Abstract

Coral reefs across the world’s oceans are in the midst of the longest bleaching event on record (from 2014 to at least 2016). As many of the world’s reefs are remote, there is limited information on how past thermal conditions have influenced reef composition and current stress responses. Using satellite temperature data for 1985–2012, the analysis we present is the first to quantify, for global reef locations, spatial variations in warming trends, thermal stress events and temperature variability at reef-scale (~4 km). Among over 60,000 reef pixels globally, 97% show positive SST trends during the study period with 60% warming significantly. Annual trends exceeded summertime trends at most locations. This indicates that the period of summer-like temperatures has become longer through the record, with a corresponding shortening of the ‘winter’ reprieve from warm temperatures. The frequency of bleaching-level thermal stress increased three-fold between 1985–91 and 2006–12 – a trend climate model projections suggest will continue. The thermal history data products developed enable needed studies relating thermal history to bleaching resistance and community composition. Such analyses can help identify reefs more resilient to thermal stress.

Record warm temperatures in recent years have been extremely stressful to coral reefs. At the time, 2014 set the record for the warmest global surface temperature. The year 2015 was 0.16 °C warmer than 2014, setting not only the record for the warmest year ever but also the record for the largest single year increase^1^. So far, 2016 has been warmer than 2015 was. Ocean warming, exacerbated by one of the strongest El Niño events on record (comparable with 1997/98 and 1982/83) on top of a general warming trend, has resulted in the longest global coral bleaching event on record. Since mid-2014, reef stakeholders (scientists, managers) have reported observations of bleached corals near-continuously and from across all three tropical ocean basins^2^. As of late-2016 the event was ongoing, with more than 40% of global reef locations having been exposed to temperature stress levels that cause bleaching (G. Liu, pers. comm.).

Reefs are among the most sensitive of all ecosystems to climate change. Stony ‘reef-building’ corals live in a symbiotic relationship with microscopic algae called zooxanthellae (*Symbiodinium spp.*), whose photosynthesis provides corals with up to 90% of their energy^3^. Environmental stressors can disrupt this relationship. The main driver of contemporary stress on coral reefs is high temperature, which together with high irradiance results in an accumulation of damage to photosystem II^4^,^5^. Under extreme stress the coral expels algae^4^,^6^, leaving its bright white aragonite skeleton visible through a thin translucent layer of coral tissue and appearing ‘bleached’. Anomalously warm sea temperatures across broad scales have been closely linked to spatially extensive ‘mass’ coral bleaching events in recent decades^7^,^8^,^9^.

There is a bleaching continuum. Some coral paling is common in many coral species during warm-season months, and bleached corals can survive mild thermal stress and recover their algae^10^. However, severely bleached corals can and have died in great numbers when exposed to persistent stressful conditions^11^,^12^. Thermally stressed corals have higher disease susceptibility^13^,^14^,^15^; and reduced reproductive output^10^ and skeletal growth^16^. Eventual impacts of bleaching (over years to decades) can include reduced reef rugosity, coral cover and biodiversity^10^; and perhaps local extinction of coral species^17^,^18^. Reefs affected by bleaching provide a lower quality habitat for fish and invertebrate species, and provide fewer ecosystem goods and services for dependent human communities^19^,^20^. As ocean waters warm under climate change, bleaching events are expected to become both more frequent and more severe^21^,^22^,^23^,^24^.

Historical temperature variation and the cumulative effects of past disturbance events influence not only the condition of reefs but also their capacity to respond to subsequent stress events^25^. Corals are known to have adapted or acclimated to local environmental conditions^26^,^27^; e.g., temperature thresholds for bleaching vary spatially and have been linked to local summertime conditions^7^. Impacts from thermal stress have been lower at sites where short-term pulses of low-level temperature stress preceded higher thermal stress later in summer^24^ or that had been affected by a prior but recent thermal stress event (e.g., Thompson & van Woesik^28^, Heron *et al*.^29^). Reef sites dominated by high-frequency variability (5.7-year period) over low-frequency variability (>54-year period) were observed to experience more intense thermal stress and severe bleaching^28^. High SST variability year-round and during the warm season has also been suggested to proffer protection for reefs from bleaching^30^,^31^,^32^. Knowledge of thermal history can shape the lens through which managers and researchers view the current condition of reefs, and how they anticipate and respond to bleaching impacts on reefs. However, until now, high-resolution spatial data on key thermal history characteristics has not been available for all global coral reef locations.

Our objective here is to assess and provide tools to understand thermal history trends and patterns for reefs worldwide at the approximate scale of reefs using 4-km SST archives. Satellite remote sensing using Advanced Very High Resolution Radiometers (AVHRR) provides the capacity to undertake analysis of sea surface temperature (SST)-based metrics over spatially vast areas at high-resolution (4 km) through recent decades. Our period of analysis, 1985–2012, spans the two previous global bleaching events confirmed to have impacted all three tropical ocean basins (i.e., global events) in 1998^11^ and 2010^33^,^34^,^35^,^36^,^37^,^38^. We quantify and compare the following metrics for all coral reef areas: (1) rates of change in annual and warm-season SST; (2) the frequency of exposure to and onset timing of bleaching-level thermal stress events; (3) the percentage of reefs exposed to bleaching-level thermal stress each year during the study period; and (4) warm-season temperature variability. Previous studies^32^,^39^,^40^,^41^,^42^,^43^ included only some of these descriptors of thermal history, were conducted at lower spatial resolution, used shorter time periods and/or were regional in nature. We present spatial analyses of these thermal history metrics globally and for the six reef regions within *Reefs at Risk–Revisited*^44^: Middle East (MID), Indian Ocean (IND), Southeast Asia (SEA), Australia (AUS), Pacific Ocean (PAC) and Atlantic Ocean (ATL). The context of future thermal exposure is included using stress projections based on the latest available global modelling.

## Results

In summary, our analysis of thermal history at global coral reef locations revealed warming at almost all reefs in recent decades; summertime temperature increased through the record at the great majority of reefs. One-third of the world’s reefs were exposed to bleaching-level thermal stress less than once per decade, with one-third of reefs exposed between once and twice per decade, and the remaining one-third exposed more than twice per decade. The global percentage of reefs impacted by bleaching stress tripled through the 28-year record, explaining the increase in observed bleaching. While the onset of thermal stress mostly coincided with the warmest part of the year, we found that at nearly one-quarter of reefs it did not. The following key points were identified from each set of thermal history parameters.

### SST Trends

**Figure 1:**
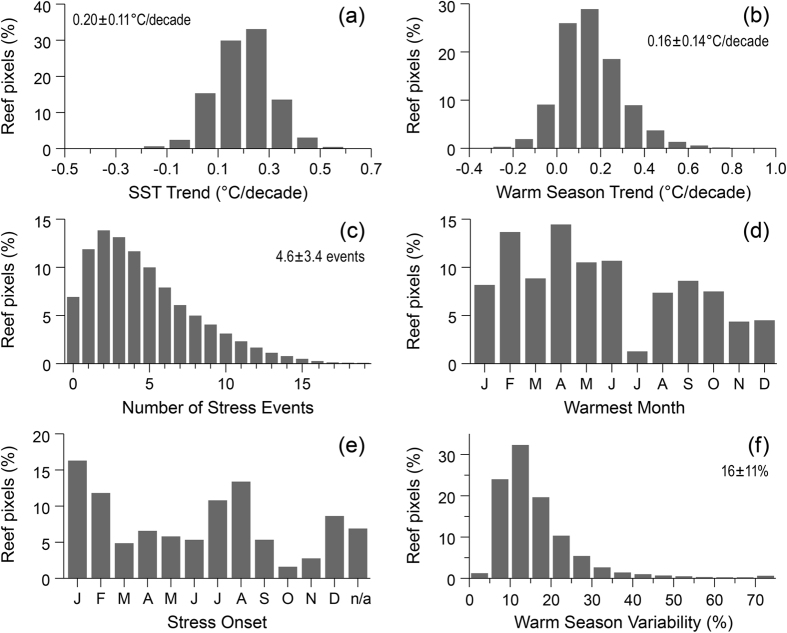
Histograms of thermal history metrics for global reef locations (n = 60,710). Global summary of the data distribution for (**a**) annual and (**b**) warm-season trends; (**c**) bleaching-level thermal stress events; (**d**) warmest month; (**e**) stress onset; and (**f**) warm-season temperature variability, 1985–2012. Warm-season temperature variability is the standard deviation of warm-season temperatures expressed as a percentage of the climatological range. Global averages ± one standard deviation are shown in plots (**a**,**b**,**c** and **f**. Data are provided for each histogram in Table S1.

**Figure 2:**
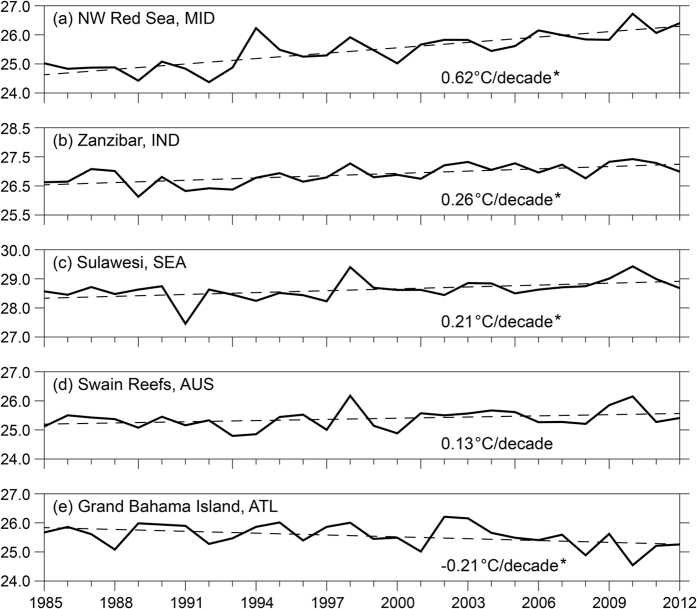
Trends in annual mean temperature at select coral reef locations. These locations approximate the 99^th^, 75^th^, 50^th^, 25^th^, and 1^st^ percentiles (**a–e**, respectively) of the annual SST Trend values in the global dataset (n = 60,710 reef pixels). Reef regions are MID = Middle East, IND = Indian Ocean, SEA = Southeast Asia, AUS = Australia and ATL = Atlantic Ocean). Trend values shown are significant (p < 0.05, denoted by*) excepting for Swain Reefs, AUS.

Coral reef SST warmed during the 28-year period, with cool seasons warming faster than warm seasons. Annual average temperature increased during the study period at nearly all 4-km reef pixels (97% or 58,847 pixels). Globally, coral reefs warmed an average of 0.20 ± 0.11 (spatial SD) °C/decade (Fig. 1a, Table S1a). Reefs across all reef regions warmed but rates varied considerably (Figure S1) with the most rapid warming (Middle East, 0.32 ± 0.13 °C/decade) nearly four times greater than the slowest (Australia, 0.08 ± 0.09 °C/decade; Table S2). The distribution of values is demonstrated by annual average temperature time-series (Fig. 2) for five reef locations for the approximately 99^th^, 75^th^, 50^th^, 25^th^ and 1^st^ percentiles for SST trend. Annual-average SST trend was positive and significant (p < 0.05) at 60% of reefs (36,308 pixels), while negative and significant at less than 0.01% of reefs (4 pixels). Locations with cooling temperatures are all in the Atlantic region north of Grand Bahama Island (Fig. 2e), which is in contrast to the general warming across the Atlantic region (0.17 ± 0.12 °C/decade).

**Figure 3:**
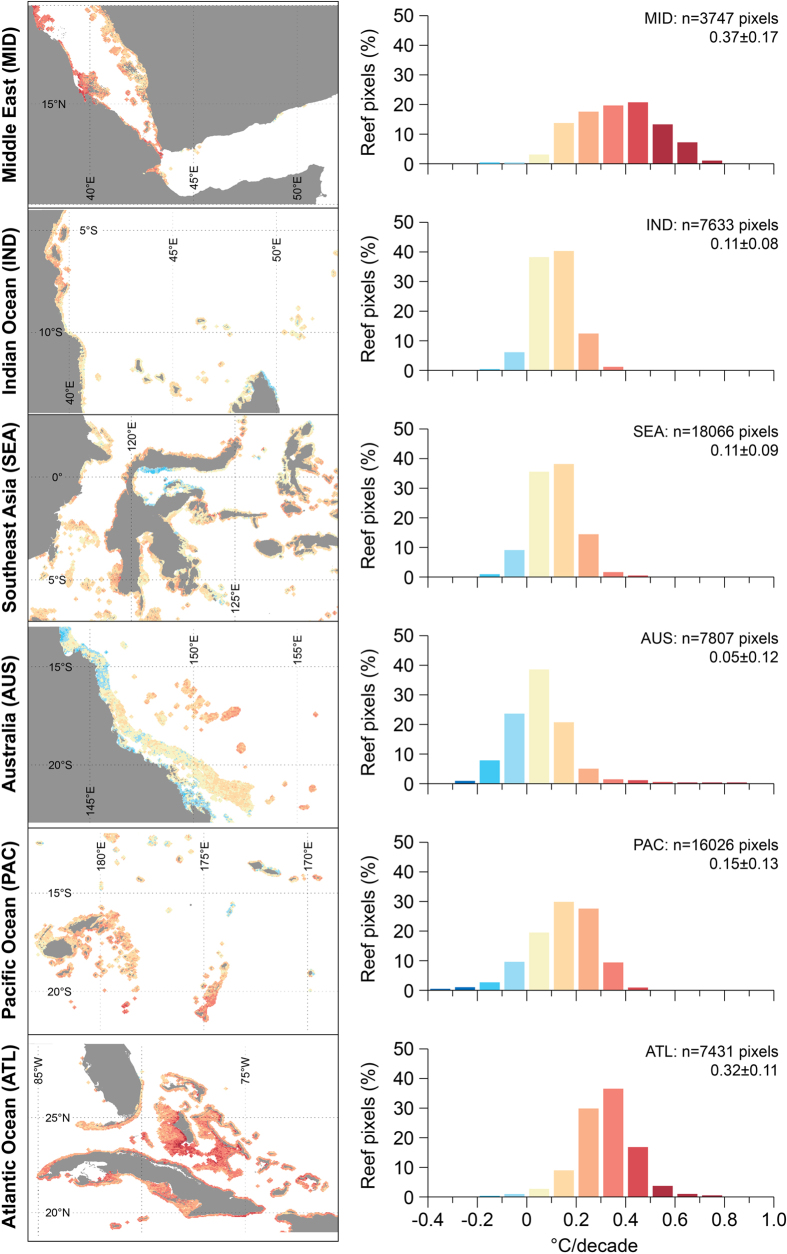
Trend in three-month warm-season temperatures among reef regions, 1985–2012. Trend values are in °C/decade. Maps (left) show results for a subset of each region; histograms (right) show the distribution of results in the full region with the regional average ± one standard deviation. Reef regions are as per Burke *et al*. 2011. Data are provided for each histogram in Table S3. Data visualisations produced using IDL [8.3] (Exelis Visual Information Solutions, Boulder, Colorado).

Bleaching stress typically occurs during the warm season. Reef SSTs warmed more slowly during the warm season (avg: 0.16 °C/decade) and had greater spatial variability (SD: 0.14 °C/decade) than the overall SST trend (Fig. 1b, Table S1b). Warm-season temperatures increased at 89% of reefs (53,768 pixels) and were significantly positive at 30% of reefs (18,362 pixels, p < 0.05). In contrast, significantly negative trends occurred at only <0.03% of reefs (16 pixels). All regional-average warm-season trends were positive (Fig. 3). Across the regions, positive trends were observed at 68–99% of reefs (Table S3). The Atlantic (92%), followed by the Middle East (47%), had the greatest percentage of reefs with statistically significant (p < 0.05) positive warm-season trends – consistent with the generally strong annual warming in these regions.

**Figure 4:**
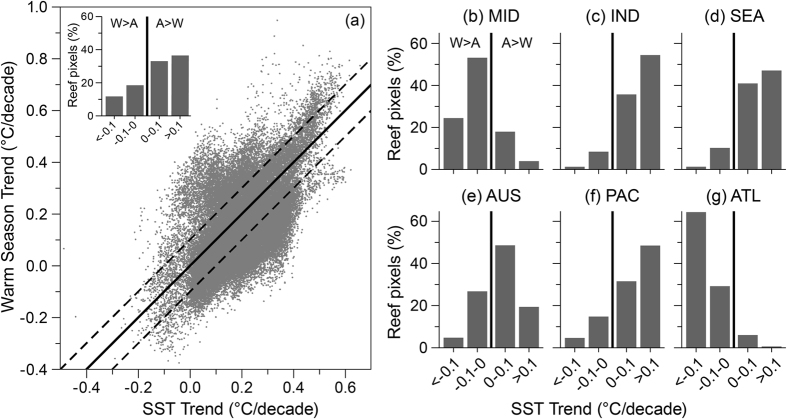
Scatterplot comparing annual SST and three-month warm-season trends globally and by reef region. Line of unity (solid) and ±0.1 °C/decade about this (dashed) are shown. Dashed lines approximate one SD of the by-pixel difference between the trends (0.11 °C/decade). Histograms show the distribution of annual SST minus warm-season trends; the solid line (at zero) corresponding to the scatterplot line of unity. Reef regions are as per Burke *et al*. 2011 (MID = Middle East, IND = Indian Ocean, SEA = Southeast Asia, AUS = Australia, PAC = Pacific Ocean and ATL = Atlantic Ocean). Data are provided for each histogram in Table S4.

Annual SST warmed faster than the warm-season trend in 70% of locations and by +0.05 °C/decade when averaged across global reefs (Fig. 4), indicating a suppression of seasonality at most reefs. The difference between annual and warm-season trends was greater than 0.1 °C/decade (~1SD about the spatial average) at 37% of the reef pixels (Table S4). The Middle East and Atlantic were the only regions where warm-season trend predominantly exceeded annual SST trend (78% for MID and 94% for ATL, see Fig. 4). In all other regions, at least 68% of the pixels had an annual SST trend greater than the warm-season trend.

### Thermal Stress

**Figure 5:**
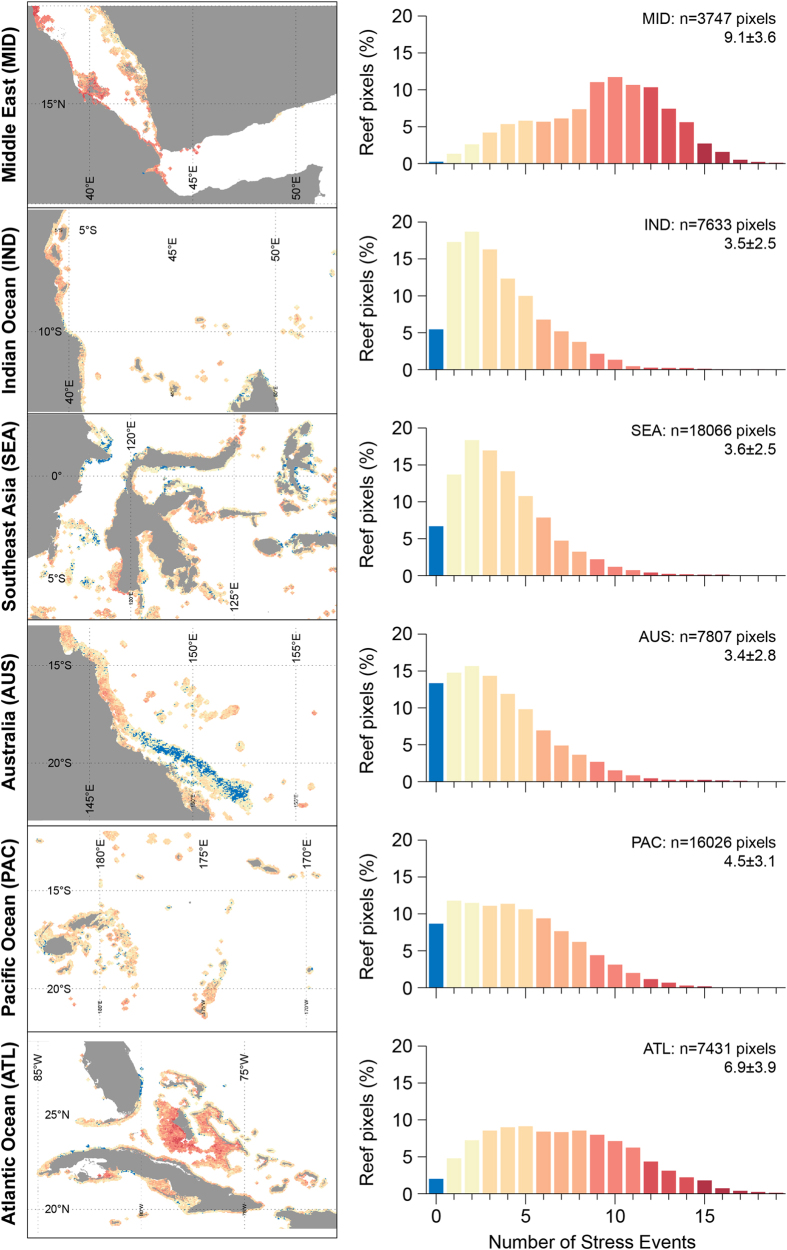
Frequency of bleaching-level thermal stress events among reef regions, 1985–2012. Bleaching-level stress is defined as DHW ≥ 4 °C-weeks. Maps (left) show results for a subset of each region; histograms (right) show the distribution of results in the full region with the regional average ± one standard deviation. Reef regions are as per Burke *et al*. 2011. Data are provided for each histogram in Table S5. Data visualisations produced using IDL [8.3] (Exelis Visual Information Solutions, Boulder, Colorado).

Most reefs (81%, n = 49,321) were exposed to multiple thermal stress events that were at a level likely to cause bleaching^12^ during the 28-year period (DHW ≥4 °C-weeks; Fig. 1c, 5). Globally, reefs were exposed to bleaching-level stress 4.6 ± 3.4 times during the 28-year study period (Fig. 1c, Table S1c), and the regional average was above three events (~1/decade) in all reef regions (Fig. 5, Table S5). Bleaching stress on reefs occurred most frequently in the Middle East (9.1 ± 3.6) and least often in the Australia region (3.4 ± 2.8). Globally, 33% of reefs (19,794 pixels) experienced bleaching-level stress two or fewer times during the record (<1/decade). The Australia, Indian Ocean and Southeast Asia regions had the highest proportions of infrequent exposure (<1/decade at 43%, 41% and 38% of reefs, respectively). One-third (33%, n = 19,831) of reefs globally experienced bleaching-level thermal stress events six or more times during the 28-year record (>2/decade), with most reefs in the Middle East and Atlantic affected (81% and 59%, respectively; Table S5, Fig. 5). Severe thermal stress (DHW ≥8 °C-weeks), linked to significant coral mortality^12^, affected 57% of global reef pixels at least once (Table S6). Just over 4% of reefs globally were exposed more than twice per decade to mortality-level thermal stress events. The Middle East and Atlantic regions had the highest proportions of reefs with frequent exposure to severe thermal stress (23% and 12%, respectively; Table S6, Figure S2).

### Temporal Patterns

**Figure 6:**
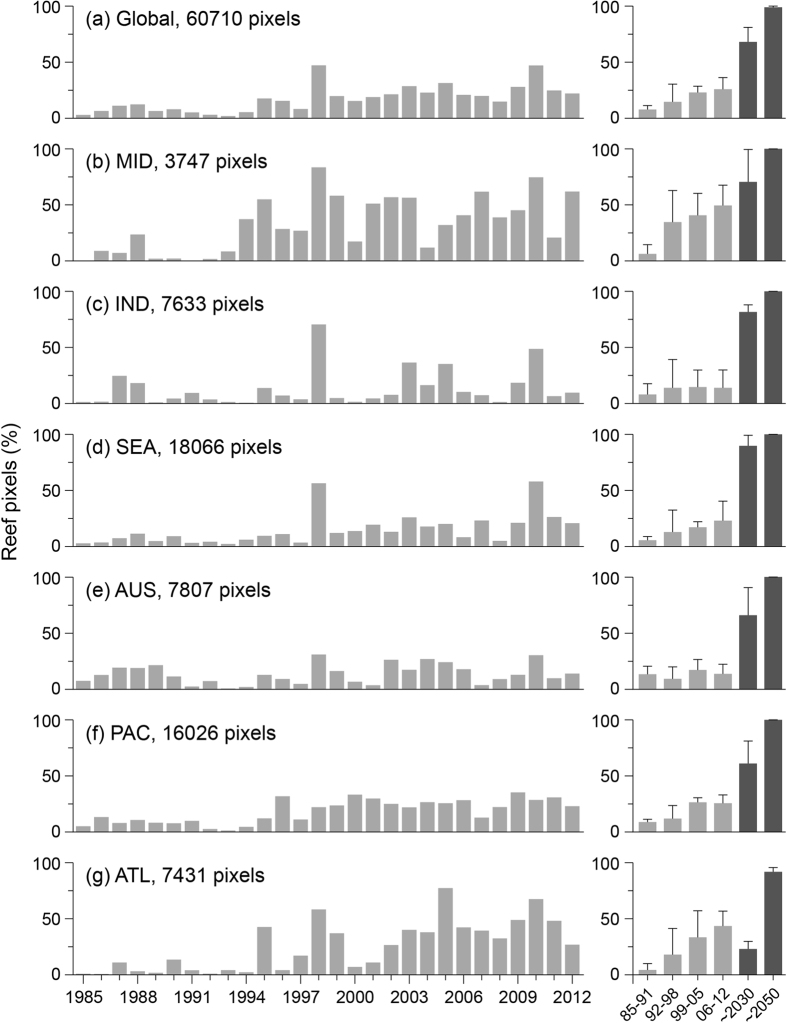
Histograms of bleaching-level thermal stress events, 1985–2012. Bleaching-level stress is defined as DHW ≥4 °C-weeks; plots refer to the % of reef pixels. The 28-year study period is divided into four 7-year periods in the histograms on the right (light grey), which show the average percentage of reef pixels affected by bleaching-level thermal stress across each period. The dark grey shade in the histograms show the average percentage of reef projected to experience bleaching-level thermal stress events under emissions scenario RCP8.5 for the 7-year periods centered on 2030 and 2050, following methods in van Hooidonk *et al*. 2014. Whiskers in the histogram are one standard deviation. Reef regions are as per Burke *et al*. 2011 (MID = Middle East, IND = Indian Ocean, SEA = Southeast Asia, AUS = Australia, PAC = Pacific Ocean and ATL = Atlantic Ocean). Data are provided for each histogram in Tables S7a,b.

In each year of 1985–2012, thermal stress was observed somewhere across global reefs (Fig. 6–left panels; Table S7a). The greatest numbers of reefs were impacted in 1998 (48%), 2010 (48%) and 2005 (32%), corresponding to the two global bleaching events and largest Caribbean bleaching event during this period. In most regions, 1998 and 2010 were the two highest ranked (either 1^st^ or 2^nd^) years for all reef regions with the exception of the Atlantic (2005, 2010) and Pacific (2009, 2000). When the record was divided into four 7-year periods, the global percentage of reef pixels that were stressed increased steadily (8, 14, 23 and 26%), tripling from 1985–91 to 2006–12 (Fig. 6–right, Table S7b). This increasing trend of bleaching-level stress events was consistent in the Middle East, Southeast Asia, Pacific and Atlantic regions. In contrast, there was no consistent temporal trend in the frequency of bleaching events in Indian Ocean and Australia reef pixels; the number of reef pixels affected in first or second 7-year period is comparable with that of the most recent period (within 1%).

The percentage of reef locations exposed to bleaching-level thermal stress events is projected by climate models^22^ to continue to increase (Fig. 6–right, Table S7b). By 2050, more than 98% of reefs are expected to be exposed to bleaching-level thermal stress in each year^21^,^22^. Even in the Atlantic region, where projections suggest reduced bleaching around 2030, more than 91% of reefs are likely to experience bleaching-level thermal stress each year by 2050 (consistent with van Hooidonk *et al*.^21^).

### Timing

Each of the calendar months was the warmest on a reef somewhere across the globe (Fig. 1c, S3). Within each region, two or three consecutive months predominated as the warmest for reefs – e.g., April-June in Southeast Asia and August-October in the Atlantic. Sub-region maps (Figure S3–left panels) indicate that geographic/oceanographic physical separation and latitudinal variation were factors defining warmest month areas. When considered globally, peaks in the onset of stress were in January/February and July/August, following the astronomical solstice events (Fig. 1e). However, many locations had onset months between solstice events, particularly reefs near the equator (in the Indian Ocean, Southeast Asia and Pacific regions; Figure S4).

Of the 93% of reefs that experienced bleaching-level thermal stress (56,521 pixels), average stress onset coincided with the warmest month at 29% (16,383) of these and occurred in the preceding 1–2 months at a further 49% (27,824) of sites. The onset of bleaching stress did not coincide with or immediately precede the warmest month in nearly one quarter of reef locations. While some of these reefs were found in each of the six regions, most were in the Southeast Asia and Pacific regions. This may reflect timing delays due to the Southeast Asian monsoon cycle and the relatively high interannual variability in the equatorial Pacific (linked to El Niño-Southern Oscillation events), respectively. Understanding spatial patterns of stress onset timing can inform managers’ preparations during broad-scale thermal events.

### Warm-season Variability

High SST variability in summer has been linked with reduced sensitivity to thermal stress^45^. However, research to date has provided no clear threshold defining “high” variability. We considered the globally most-variable locations (approximately the upper quartile) as having high variability and examined the distribution of these reefs. Nearly one-quarter of global reefs (23%) had warm-season variability at or above 20% of the climatological range (Fig. 1f). There were no high-variability reef pixels in the Middle East, and very few in Australia (1%) and the Atlantic (9%); in contrast, between one- and two-thirds of reef pixels in Southeast Asia, the Indian Ocean and the Pacific Ocean were among the most variable (Figure S5, Table S8). Greater exposure to variable warm-season temperature may be important in stimulating adaptive responses in corals^46^.

## Discussion

Temperature trends indicate accelerated warming in recent decades. Overall, reefs have been increasingly exposed to bleaching stress through this period. However, when comparing changes in exposure frequency across the record with the local summertime warming trend, some reefs experienced a lower-than-expected increase to stress exposure based on the global pattern, suggesting these locations as potential refugia. Using our analysis we identify reefs potentially more resilient to climate change impacts to inform conservation efforts.

### SST Trends

Warming of coral reef waters (Fig. 1a, S1) was distinctly higher than that reported for ocean waters in general, both globally (0.10–0.12 °C/decade, 1971–2010^47^,^48^) and regionally (0.02–0.13 °C/decade, 1950–2009^23^). Consistent with IPCC findings, warming in the Indian Ocean (from the Middle East and Indian Ocean regions) exceeded that in the Pacific (from the Southeast Asia, Australia and Pacific Ocean regions), which in turn was greater than that in the Atlantic. Higher trends on reefs likely reflect the accelerated rate of warming from the most recent 28-year period (compared with the longer timeframes used in IPCC analyses), and potentially result from better resolution and improved accuracy of data closer to land^39^. Regional trends in annual and warm-season temperature (Fig. 3, S1) were consistent with earlier studies in the Atlantic^40^,^41^ and in Southeast Asia^42^.

Warming trends vary broadly across reefs – annual average temperature in the northwestern Red Sea, Middle East (99^th^ percentile) increased at approximately three times the global average. In contrast, temperature to the north of Grand Bahama Island in the Atlantic (1^st^ percentile) declined at −0.21 °C/decade – comparable to the rate of average global increase (Fig. 2). Recent cooling observations in parts of the Atlantic region have been linked to an increase in winter cold-air fronts from the North American continent since the 1990s^49^, including unusually cold weather causing coral mortality in Florida in 2010^50^. Warm-season trends in the Atlantic region were predominantly greater than annual SST trends (Fig. 4g), consistent with Chollett *et al*.^41^. Warm-season warming may have been driven by the negative- to positive-phase change of the Atlantic Multidecadal Oscillation around the mid-to-late 1990s^51^, also linked to increased oceanic heat content and Atlantic tropical storm activity in recent years^52^.

Faster warming in winter than in summer for 70% of global reefs (Fig. 4) is consistent with both observations through the past century and future predictions that winter temperatures are warming faster than summer temperatures^53^,^54^. The consequence for corals has been a steady reduction in the cool-season reprieve from warm-season temperatures, which can enhance disease outbreaks^55^,^56^. In contrast, reefs experiencing more rapid warming of their warm seasons may experience increased bleaching and infectious disease^13^,^14^,^15^.

### Thermal Stress

Reefs with infrequent bleaching stress events (DHW ≥4 °C-weeks, <1/decade; Fig. 1c, 5) would likely, all else being equal, have had sufficient time to recover between events^10^,^57^. This applies to 33% of reef pixels worldwide and to more than 41% of the pixels in the Indian Ocean and Australia regions, but to far fewer in the Atlantic (14%) and Middle East (4%). While low past exposure does not guarantee future refuge from stress, it can indicate localised features (e.g., upwelling) that, if these persist, may provide some protection from thermal stress^58^,^59^. Alternatively, such locations may simply have been ‘lucky so far’ in escaping exposure to stress^60^.

In contrast, reefs with high frequency of bleaching-level exposure (>2/decade) may have impaired function or may have already experienced shifts from susceptible to tolerant coral communities^10^,^57^. This applied to 33% of reefs globally and >59% in the Middle East and Atlantic regions, but <21% of reefs in the Indian Ocean, Southeast Asia and Australia regions. Corals that have survived past frequent bleaching stress events may be among the hardier and more resistant species or may have acclimated to stressful conditions^28^. Such reefs may be the most likely to persist when exposed to future stress events, though probably with the cost of reduced species and genetic diversity of surviving corals (e.g., refs 10, 29 and 57). Reefs that have persisted despite frequent exposure to mortality-level stress (DHW ≥8 °C-weeks, >2/decade) may prove critical for the continued existence of corals into the future. Nearly 24% of global reef locations (n = 14,672) experienced mortality-level thermal stress in one or both of 1998 and 1999, suggesting that the reported 16% loss of reefs^11^ from the first recorded global bleaching may have been substantially underestimated.

Temporal patterns in bleaching-level thermal stress (Fig. 6) show that reefs have been increasingly exposed to stress in recent decades, with variation across the regions. Dramatic increases in the regional percentage of stressed reefs were likely associated with switches in basin-scale oceanographic phenomena: in the Middle East region during 1992–98, coinciding with the switch in the Indian Ocean Dipole^61^ and resulting in increased bleaching^62^; and in the Pacific region during 1999–2005, following the ca. 1998 phase shift in the Pacific Decadal Oscillation^63^.

**Figure 7:**
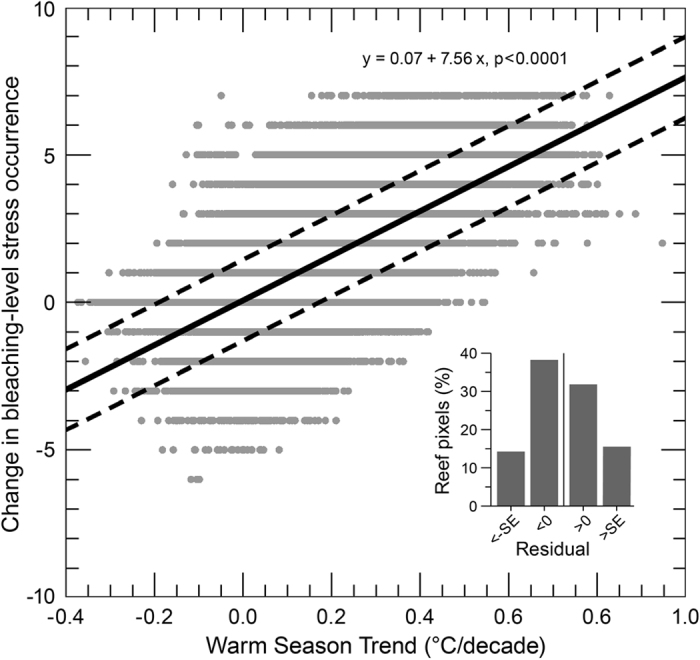
Change in bleaching stress occurrence with warm-season temperature trend. Difference in number of bleaching-level stress events (DHW ≥4 °C-weeks) between the 1985–1991 and 2006–2012 periods compared with warm-season trend for global reef pixels. Solid line shows linear regression; dashed lines are one standard error of estimate (SE = 1.37) above and below this. Histogram shows the proportions of reef pixel residuals distinguished by the lines. Data are provided for the histogram in Table S9.

Our assessment of both trends and stress exposure provides, for the first time, the opportunity to examine how these interact. We evaluated how summertime warming rate affected the frequency of bleaching stress events. The increase of bleaching-level events from 1985–91 to 2006–12 was associated with warm-season warming for many reefs (Fig. 7). For over 14% of reefs (8,704 pixels, Table S9), however, the change in stress exposure was more than one SE below the global linear regression. These reefs, present in all regions, had less increase to exposure than expected given their summertime warming rate.

### Warm-season Variability

Several field studies^45^,^46^,^64^,^65^ show that higher temperature variability reduces susceptibility to thermal stress on local scales; however, no variability threshold for or quantitative relationship with the mitigation of stress has been defined. To examine this, we considered the warm-season variability values at the reefs from these studies. With the exception of the study of Oliver & Palumbi^45^, each spanned multiple pixels. No absolute threshold value could be ascribed to distinguish sites (e.g., the “low variability” location from Carilli *et al*.^65^ had a metric value greater than that of the “high variability” location in Castillo *et al*.^64^). This suggests that spatial patterns in temperature variability on a regional-to-local scale may be more important than a global threshold in identifying reefs resistant to thermal stress. The warm-season temperature variability data product enables broad-scale studies to test the hypothesis that high temperature variability reduces bleaching impacts^66^.

### Application to Conservation

Identifying reefs with reduced exposure and/or less sensitivity can assist in identifying short-term target locations for conservation, which is critical given that projections of future bleaching indicate near-complete exposure of reefs to annual bleaching-level stress around 2050 (Fig. 6a–right, van Hooidonk *et al*.^21^). The production of the maps and spatial data presented here creates an opportunity to test hypotheses of how bleaching impact may be influenced by thermal history. With the third global coral bleaching event in progress at the time of writing, observations from this event can be used to validate how aspects of thermal history influence the severity of bleaching responses and levels of bleaching-induced mortality.

Here, we consider three characteristics of thermal history to identify reefs potentially resilient to thermal stress: (i) the frequency of past exposure; (ii) how that frequency has changed in the context of warm-season trend; and (iii) the level of warm-season variability. For each, we provide high-resolution images of identified reefs to inform conservation and research efforts (Figures S6–S8).

There is potential for both low and high frequency of past thermal exposure to be important for conservation (Figure S6). Regions with low historical exposure (blue), which are potential thermal refugia, include the Maldives and the southern Great Barrier Reef. Those with high exposure (red), which may have developed resistance, include Zanzibar and the Meso-American Barrier Reef. Some areas had both low and high exposure reefs within tens of kilometres (e.g., New Caledonia, the Florida Keys). Magris *et al*.^40^ identified reefs in southern Brazil as historical refugia due to relatively low past thermal exposure (among Brazilian reefs); our study found several reefs in this region that experienced relatively low exposure frequency (Figure S6).

Reefs with a lower increase in stress exposure (the number of bleaching stress events) than expected from their summertime warming rate (i.e., reefs with large negative residuals in Fig. 7) are potential refugia. While it is unknown if this may continue into the future, this characteristic warrants consideration of these sites as priorities for management action (Figure S7). This trait was apparent at reefs in the eastern Persian Gulf, the northern Great Barrier Reef, New Caledonia and around the Bahamas and Greater Antilles. Maina *et al*.^67^ identified reefs along the southern African coast and east of Madagascar as among western Indian Ocean reefs with the lowest susceptibility to thermal stress; reef locations in this region were also identified in Figure S7.

Reef locations with the highest observed warm-season variability (≥20% of the climatological range) were found in the Maldives, western Sumatra, the Solomon Islands and Micronesia, several islands in the south Pacific, and the Caribbean coast of Panama (Figure S8). Given the lack of information on a threshold for warm-season variability, we propose that these reefs, which have the highest variability globally, be considered as priority conservation sites. However, consideration might also be given to the most variable reefs within individual regions/sub-regions.

Reefs with slower future warming could also be valuable sites for conservation. These can be identified globally once downscaled model projections, such as those for the Caribbean presented in van Hooidonk *et al*.^68^, are available for all reef regions.

Understanding the capacity of corals to cope with thermal stress exposure may be the most important factor in predicting future reef trajectories^69^. Guided by remote sensing products that monitor thermal stress in near real-time^29^,^70^ and modelled seasonal outlooks providing up-to-four-month advance warming^71^, observers are surveying reef impacts across global reef regions. The thermal history data products described here (and available at: http://coralreefwatch.noaa.gov/satellite/thermal_history/) enable studies relating thermal history to bleaching resistance and community composition. Such analyses are needed, especially in light of thermal exposure during the current global event, to expand on the efforts presented here in helping identify reefs more resilient to thermal stress.

## Conclusion

This study is the most comprehensive retrospective analysis of sea surface temperature and historical thermal stress in coral reef areas undertaken to date. Results from 1985–2012 show that: (i) 97% of reef pixels warmed through this period; (ii) cooler seasons represented less of a reprieve from warm-season stress; and (iii) more than three times as many reef pixels were exposed to bleaching-level thermal stress at the end of the record than was characteristic of the late 1980s, with even more drastic increases expected in coming decades. Importantly, the spatial heterogeneity seen in the analysis may identify locations that either represent refugia, or have reduced sensitivity to thermal stress and which could be less impacted during future disturbance. Coral bleaching events have become and will continue to become more frequent and severe – it is critical that we identify and conserve resilient reefs to help coral reefs survive while efforts are underway to control damaging anthropogenic global warming.

## Methods

We used the NOAA Pathfinder version-5.2 daily, 1/24° (~4 km) sea surface temperature (SST) dataset, derived from satellite remote sensing and an official NOAA Climate Data Record for SST^72^. This latest version of Pathfinder provides continuous and consistently derived reef-scale temperatures over recent decades, currently available through 2012. This product provides skin temperature whilst previous versions of Pathfinder reported bulk temperatures, which have an average offset of ~0.16 °C^73^. This offset can vary with wind speed, cloud cover and other atmospheric parameters^73^. However, the internal consistency of this dataset and the fact that it spans both previous global coral bleaching events in 1998 and 2010 (see introduction) are key considerations supporting its use. Pathfinder SST data were composited to weekly resolution and then gap-filled using temporal and spatial-comparison techniques for 1985–2012 following Heron *et al*.^55^.

We assessed which pixels contain coral reefs by combining three published global reef-locations datasets (ReefBase^74^, Millennium Maps^75^, Reefs at Risk–Revisited^44^,^76^). This was further augmented by other documented coral reef locations from collaborative reef studies; the reef-pixel dataset is available at coralreefwatch.noaa.gov. SST analysis was performed for 60,710 reef-containing pixels; maps presented here include pixels within ~9 km of reefs (total n = 175,585) to enhance visual interpretation of the results.

A range of thermal history metrics was developed in consultation with reef scientists and managers and arranged into six themes: 1. Trends (SST rates of change); 2. Climatology (long-term average conditions); 3. SST Variability (seasonal and annual); 4. Annual History (maximum SST, anomaly and DHW, by year); 5. Stress Frequency (number of events for different stress levels); and 6. Onset Timing (expected onset and variability). From within these six themes, this study focused on seven metrics (numbered below).

*SST trend* in temperature [metric 1a] provides the long-term (28-year) historical trajectory of annual-mean temperature (*SST*_*ann*_) as the slope, *ω*_*ann*_, of a linear generalised least squares model (after Weatherhead *et al*.^77^):


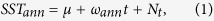


where *μ* is constant, *t* is time in years and *N*_*t*_ is the residual assumed to be autoregressive of the order of 1. The residual at a given time is a linear function of the residual at the previous time step and a random variable, *ε*_*t*_ (i.e., *N*_*t*_  =  ϕ *N*_*t*−*1*_ + *ε*_*t*_). Statistical significance of the trends was determined at the 5% level (i.e., p < 0.05). To ensure appropriate representation of global coral reef regions (see below), regional results were compiled for all trends as well as the subset that were statistically significant.

Expected intra-annual temperature variations on reefs can be described by long-term monthly averages (climatologies), developed here following Heron *et al*.^78^. The warmest of these, the Maximum of the Monthly Means (MMM, °C), is used by NOAA Coral Reef Watch (CRW) as the stress threshold for monitoring conditions conducive to bleaching^70^. The climatologically *warmest month* [2] varies across global reef locations and indicates the period when bleaching-level thermal stress is most likely.

To examine factors during the intra-annual period when there is potential for coral bleaching, we defined the three-month warm season as centred on the warmest month for each pixel. *Warm-season trend* in temperature [1b] is the slope, *ω*_*ws*_, of the generalised least squares model with autoregressive covariance (order 1) during the thermal stress period, calculated using three-month average temperature for the warm season, *SST*_*ws*_, within each year, as:


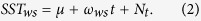


Parameters and statistical significance for warm-season trend are as described for the annual SST trend. The difference between SST trend and warm-season trend provides an indication of how the seasonality may have increased, or become suppressed, through the record. Reef locations for which SST trend exceeds warm-season trend indicate a suppressed seasonality, and therefore less respite from summertime temperature. Locations with a marked reduction in seasonality were identified by calculating the standard deviation of the trend difference across global reef pixels, and then determining where the trend difference was greater than approximately one standard deviation.

Bleaching-level thermal stress was calculated using Degree Heating Weeks (DHW), which combines magnitude and duration of temperature exceeding the MMM^70^. DHW of 4 °C-weeks has been linked to ecologically significant coral bleaching^12^ and was used here to indicate bleaching-level thermal stress. DHW of 8 °C-weeks is associated with significant coral mortality^12^ and was used as the threshold for mortality-level thermal stress. Knowledge of the likely onset timing of the bleaching season, and the spatial context of this information, can assist reef stakeholders in long-term planning, short-term preparation and monitoring. The mean timing of *stress onset* [3] (month) for thermal stress that reached 4 °C-weeks or greater was documented for all locations. Information on annual historical exposure can guide managers in understanding past thermal stress; identifying local DHW thresholds (i.e., if different from the broadly used values of 4 and 8 °C-weeks^12^); and distinguishing between thermal and non-thermal bleaching events. *Annual maximum DHW* [4] provides the highest accumulated thermal stress in each year. We quantified the *number of bleaching-level stress events* [5] through the 28-yr record, describing the historical incidence of annual maximum DHW at or above DHW of 4 °C-weeks. Reefs that experienced bleaching-level stress with frequency <1 event/decade (two or fewer occurrences) were defined as having had relatively low frequency of exposure to thermal stress, while those with >2 events/decade (six or more occurrences) were defined as having high frequency of exposure (see Donner *et al*.^57^ and discussion therein). The number of mortality-level stress events through the record was determined using the DHW threshold of 8 °C-weeks.

The spatial distribution of the potential for increased thermal tolerance due to temperature variability was evaluated by defining *warm-season variability* [6], the standard deviation around the long-term mean of three-month warm-season temperature. This metric was calculated following the removal of the warm-season trend to separate the effects of long-term change and variability. Previous global mapping of SST variability at reduced resolution (0.5–1.0°) indicated strong influence of latitudinal variation^32^. To eliminate the effect of latitudinal variation, the warm-season variability was expressed here as a percentage of the climatological temperature range; i.e., the difference between the maximum (MMM) and minimum of the monthly mean climatologies. As the level of variability that confers bleaching resistance is unknown, we identified locations where the variability scaled by the local climatological range was in the approximately upper quartile of global values.

Spatial analysis of the aforementioned metrics was undertaken for reef locations globally (n = 60,710). To provide further insight into regional (ocean basin/sub-basin) patterns of the metrics while aligning with existing conservation management knowledge, spatial analyses were also undertaken for the six regions defined by the World Resources Institute’s Reefs at Risk–Revisited analysis^44^. These reef regions are as follows: Middle East (MID), Indian Ocean (IND), Southeast Asia (SEA), Australia (AUS), Pacific Ocean (PAC) and Atlantic Ocean (ATL). Global and regional summaries for each metric were calculated as the average and standard deviation (SD) across reef pixels within these regions and globally. Maps from within each region in the main text and supplementary material display within-region variation for each metric. The areas shown are centred on: the southern Red Sea (Middle East), Comoros (Indian Ocean), Sulawesi (Southeast Asia), the central Great Barrier Reef (Australia), Fiji/Samoas (Pacific Ocean) and the Bahamas (Atlantic Ocean). Distributions of data from reef locations globally and from across each region are presented as histograms, and the corresponding data are provided in the supplementary material. Time-series and trends of annual-mean SST are displayed for five reef locations (across five of the six regions) that represent the ~99^th^, ~75^th^, ~50^th^, ~25^th^ and ~1^st^ percentiles of annual SST trend.

Temporal patterns in the historical incidence of bleaching- and mortality-level stress were considered annually and by dividing the 28-year period into four 7-year periods (1985–91, 1992–98, 1999–2005, 2006–12). Global and regional historical patterns were augmented using projections of thermal stress (DHW), based on monthly SST data from the World Climate Research Programme’s Coupled Model Intercomparison Project Phase 5 (CMIP5) dataset^79^. Projected stress was calculated from 33 available GCMs under relative concentration pathway (RCP) 8.5 following the methods presented in van Hooidonk *et al*.^21^ (model list in van Hooidonk *et al*.^22^). For the projections, the equivalent DHW value for bleaching-level thermal stress was 6 °C-weeks^21^. The median year for the start of annual bleaching conditions under RCP8.5 was reported as 2040^21^. To allow comparison with temporal patterns from the 7-year periods in the historical satellite data, we calculated the percentage of reef pixels with bleaching-level stress across 7-year periods centred on 2030 and 2050 (10 years prior and subsequent to the reported median year). We present regionally summarised information of projected thermal stress for comparisons with the four 7-year periods between 1985 and 2012; projections at full resolution are in refs 21, 22 and 56.

Remote sensing data collation, spatial analysis and data visualization was undertaken using Interactive Data Language (IDL) v8.1–3 and python 2.7.

## Additional Information

**How to cite this article**: Heron, S. F. *et al*. Warming Trends and Bleaching Stress of the World’s Coral Reefs 1985–2012. *Sci. Rep.* **6**, 38402; doi: 10.1038/srep38402 (2016).

**Publisher's note:** Springer Nature remains neutral with regard to jurisdictional claims in published maps and institutional affiliations.

## Supplementary Material

Supplementary Information
