# Supplementary material for: Warming Trends and Bleaching Stress of the World’s Coral Reefs 1985–2012
Source: Sci Rep. 2016 Dec 6;6:38402. doi: 10.1038/srep38402 (PMC5138844; doi:10.1038/srep38402)
Supplement: Supplementary Information [file srep38402-s1.pdf]

Supplementary Material: ***Warming Trends and Bleaching Stress of the World's Coral Reefs 1985-2012***, SF Heron, JA Maynard, R van Hooidonk, CM Eakin. *Note: Throughout the supplementary material, reef regions are as per Burke et al. (2011)*[1].

Table S1. Global summary of the data distribution for (a) annual and (b) warm-season trends; (c) bleaching-level thermal stress events; (d) warmest month; (e) stress onset; and (f) warm-season temperature variability, 1985-2012. Warm-season temperature variability is the standard deviation of warm-season temperatures expressed as a percentage of the climatological range. These data correspond to histograms presented in Figure 1a-f. Data distribution among reef regions for these three variables is presented in Tables S2-3, S5, S8, S10-11.

	(a) **SST trend (°C/decade)**		(b) **Warm-season trend (°C/decade)**	
°C/decade	Pixels	%	Pixels	%
Average±SD (% significant)	*0.20±0.11 (60%)*		*0.16±0.14 (30%)*	
-0.5 to -0.4	2	0.0	0	-
-0.4 to -0.3	0	-	27	0.0
-0.3 to -0.2	13	0.0	193	0.3
-0.2 to -0.1	356	0.6	1170	1.9
-0.1 to 0.0	1492	2.5	5552	9.1
0.0 to 0.1	9471	15.6	15838	26.1
0.1 to 0.2	18374	30.3	17629	29.0
0.2 to 0.3	20369	33.6	11299	18.6
0.3 to 0.4	8382	13.8	5459	9.0
0.4 to 0.5	1902	3.1	2278	3.8
0.5 to 0.6	342	0.6	813	1.3
0.6 to 0.7	7	0.0	357	0.6
0.7 to 0.8	0	-	87	0.1
0.8 to 0.9	0	-	7	0.0
0.9 to 1.0	0	-	1	0.0

Trends shading: light grey denotes negative values; dark grey denotes values that exceeded the global average plus one SD (~0.30 °C/decade for both annual SST and warm-season trends).

	(c) **Bleaching-Level thermal stress events (#)**	
#	Pixels	%
Average±SD	*4.6±3.4*	
0	4185	6.9
1	7204	11.9
2	8405	13.8
3	7960	13.1
4	7078	11.7
5	6047	10.0
6	4795	7.9
7	3674	6.1
8	3022	5.0
9	2458	4.0
10	1886	3.1
11	1389	2.3
12	1004	1.7
13	669	1.1
14	455	0.7
15	273	0.4
16	127	0.2
17	47	0.1
18	26	0.0
19	6	0.0

Events shading: light grey denotes <1 event/decade on average; dark grey denotes >2 events/decade.

[1] Burke, L., Reytar, K., Spalding, M., & Perry, A. *Reefs At Risk Revisited* (World Resources Institute, 2011).

Table S1. Global summary of the data distribution for (a) annual and (b) warm-season trends; (c) bleaching-level thermal stress events; (d) warmest month; (e) stress onset; and (f) warm-season temperature variability, 1985-2012. Warm-season temperature variability is the standard deviation of warm-season temperatures expressed as a percentage of the climatological range. These data correspond to histograms presented in Figure 1a-f. Data distribution among reef regions for these three variables is presented in Tables S2-3, S5, S8, S10-11.

(d) **Warmest month**		
Month	Pixels	%
January	4967	8.2
February	8311	13.7
March	5358	8.8
April	8776	14.5
May	6388	10.5
June	6508	10.7
July	792	1.3
August	4475	7.4
September	5219	8.6
October	4542	7.5
November	2649	4.4
December	2725	4.5

(e) **Stress onset (month)**		
Month	Pixels	%
January	9889	16.3
February	7191	11.8
March	2981	4.9
April	3983	6.6
May	3501	5.8
June	3236	5.3
July	6540	10.8
August	8173	13.5
September	3196	5.3
October	948	1.6
November	1655	2.7
December	5228	8.6
[no event]	4189	6.9

(f) **Warm-season variability (% climatological range)**		
%	Pixels	%
Average±SD	*16±11*	
0-5	747	1.2
5-10	14540	23.9
10-15	19562	32.2
15-20	11938	19.7
20-25	6235	10.3
25-30	3285	5.4
30-35	1553	2.6
35-40	862	1.4
40-45	609	1
45-50	392	0.6
50-55	296	0.5
55-60	152	0.3
60-65	96	0.2
65-70	63	0.1
70+	380	0.6

Warm-season variability shading: light grey denotes the approximately one- quarter of sites with highest variability (>20%).

Table S2. Regional data distribution for trend in annual temperatures (°C/decade), 1985-2012. These data correspond to the histograms shown on the right side of Figure S1. Negative values (temperatures decreased during the study period) are shown in light grey shade, and values greater than the global average plus one standard deviation (0.20 + 0.10 °C/decade) are in dark grey shade.

**SST trend** °C/decade	**Middle East** Pixels	%	**Indian Ocean** Pixels	%	**Southeast Asia** Pixels	%	**Australia** Pixels	%	**Pacific Ocean** Pixels	%	**Atlantic Ocean** Pixels	%
Average±SD (% significant)	*0.32±0.13 (47%)*		*0.21±0.05 (84%)*		*0.20±0.07 (79%)*		*0.08±0.09 (8%)*		*0.24±0.12 (58%)*		*0.17±0.12 (55%)*	
-0.5 to -0.4	2	0.1	0	-	0	-	0	-	0	-	0	-
-0.4 to -0.3	0	-	0	-	0	-	0	-	0	-	0	-
-0.3 to -0.2	0	-	0	-	0	-	0	-	11	0.1	2	0.0
-0.2 to -0.1	2	0.1	0	-	0	-	144	1.8	185	1.2	25	0.3
-0.1 to 0.0	14	0.4	0	-	17	0.1	596	7.6	478	3.0	387	5.2
0.0 to 0.1	137	3.7	69	0.9	1193	6.6	4482	57.4	1707	10.7	1883	25.3
0.1 to 0.2	731	19.5	3209	42.0	7621	42.2	2135	27.3	2312	14.4	2366	31.8
0.2 to 0.3	726	19.4	4050	53.1	7888	43.7	182	2.3	5862	36.6	1661	22.4
0.3 to 0.4	1003	26.8	305	4.0	1313	7.3	119	1.5	4924	30.7	718	9.7
0.4 to 0.5	943	25.2	0	-	33	0.2	147	1.9	454	2.8	325	4.4
0.5 to 0.6	188	5.0	0	-	1	0.0	2	0.0	91	0.6	60	0.8
0.6 to 0.7	1	0.0	0	-	0	-	0	-	2	0	4	0.1

Table S3. Regional data distribution for trend in warm-season temperatures (°C/decade), 1985-2012. These data correspond to the histograms shown on the right side of Figure 3. Negative values (temperatures decreased during the study period) are shown in light grey shade, and values greater than the global average plus one standard deviation (0.16 + 0.14 °C/decade) are in dark grey shade.

**WS trend**	**Middle East**		**Indian Ocean**		**Southeast Asia**		**Australia**		**Pacific Ocean**		**Atlantic Ocean**	
(°C/decade)	Pixels	%	Pixels	%	Pixels	%	Pixels	%	Pixels	%	Pixels	%
Average±SD (% significant)	*0.37±0.17 (47%)*		*0.11±0.08 (12%)*		*0.11±0.09 (21%)*		*0.05±0.12 (5%)*		*0.15±0.13 (29%)*		*0.32±0.11 (92%)*	
-0.4 to -0.3	0	-	0	-	0	-	1	0.0	26	0.2	0	-
-0.3 to -0.2	1	0.0	0	-	0	-	63	0.8	129	0.8	0	-
-0.2 to -0.1	17	0.5	30	0.4	108	0.6	606	7.8	404	2.5	5	0.1
-0.1 to 0.0	18	0.5	486	6.4	1630	9.0	1849	23.7	1518	9.5	51	0.7
0.0 to 0.1	127	3.4	2938	38.5	6443	35.7	3018	38.7	3125	19.5	187	2.5
0.1 to 0.2	529	14.1	3093	40.5	6927	38.3	1633	20.9	4791	29.9	656	8.8
0.2 to 0.3	673	18.0	974	12.8	2629	14.6	390	5.0	4421	27.6	2128	29.8
0.3 to 0.4	750	20.0	109	1.4	287	1.6	102	1.3	1492	9.3	2719	36.6
0.4 to 0.5	791	21.1	3	0.0	39	0.2	88	1.1	112	0.7	1245	16.8
0.5 to 0.6	510	13.6	0	-	3	0.0	29	0.4	8	0.0	263	3.5
0.6 to 0.7	281	7.5	0	-	0	-	13	0.2	0	-	63	0.8
0.7 to 0.8	49	1.3	0	-	0	-	11	0.1	0	-	27	0.4
0.8 to 0.9	1	0.0	0	-	0	-	3	0.0	0	-	3	0.0
0.9 to 1.0	0	-	0	-	0	-	1	0.0	0	-	0	-

Table S4. Regional differences between the annual and warm-season trends, 1985-2012. The line separating the second and third rows corresponds to the line of unity in Figure 4; values above refer to pixels where the warm-season trend is greater than the annual trend and values below refer to pixels where the annual trend is greater than the warm-season trend.

**Trend difference**	**Global**		**Middle East**		**Indian Ocean**		**Southeast Asia**		**Australia**		**Pacific Ocean**		**Atlantic Ocean**	
(°C/decade)	Pixels	%	Pixels	%	Pixels	%	Pixels	%	Pixels	%	Pixels	%	Pixels	%
≤-0.1	7177	11.8	923	24.6	90	1.2	258	1.4	377	4.8	744	4.6	4785	64.4
-0.1-0	11195	18.4	1999	53.3	656	8.6	1889	10.5	2091	26.8	2395	14.9	2165	29.1
0-0.1	20134	33.2	678	18.1	2725	35.7	7400	41.0	3806	48.8	5081	31.7	444	6.0
≥0.1	22204	36.6	147	3.9	4162	54.5	8519	47.2	1533	19.6	7806	48.7	37	0.5

Table S5. Frequency of bleaching-level thermal stress events, 1985-2012. These data correspond to histograms presented in Figure 5. Bleaching-level stress defined as DHW ≥ 4 °C-weeks. Light-grey shade denotes pixels that experienced 0-2 events (<1/decade) on average, and dark-grey shade denotes pixels that experienced ≥6 events (>2/decade) on average.

**Number of events**	**Middle East**		**Indian Ocean**		**Southeast Asia**		**Australia**		**Pacific Ocean**		**Atlantic Ocean**	
	Pixels	%	Pixels	%	Pixels	%	Pixels	%	Pixels	%	Pixels	%
Average±SD	*9.1±3.6*		*3.5±2.5*		*3.6±2.5*		*3.4±2.8*		*4.5±3.1*		*6.9±3.9*	
0	8	0.2	413	5.4	1192	6.6	1034	13.2	1388	8.7	150	2.0
1	50	1.3	1319	17.3	2448	13.6	1145	14.7	1888	11.8	354	4.8
2	97	2.6	1423	18.6	3299	18.3	1212	15.5	1836	11.5	538	7.2
3	157	4.2	1242	16.3	3037	16.8	1112	14.2	1777	11.1	635	8.5
4	199	5.3	937	12.3	2532	14.0	920	11.8	1821	11.4	669	9.0
5	216	5.8	760	10.0	1931	10.7	758	9.7	1705	10.6	677	9.1
6	212	5.7	516	6.8	1400	7.7	536	6.9	1510	9.4	621	8.4
7	227	6.1	393	5.1	834	4.6	374	4.8	1229	7.7	617	8.3
8	275	7.3	281	3.7	563	3.1	276	3.5	992	6.2	635	8.5
9	413	11.0	160	2.1	384	2.1	203	2.6	708	4.4	590	7.9
10	438	11.7	100	1.3	207	1.1	111	1.4	500	3.1	530	7.1
11	398	10.6	33	0.4	117	0.6	59	0.8	318	2.0	464	6.2
12	386	10.3	19	0.2	63	0.3	30	0.4	186	1.2	320	4.3
13	277	7.4	13	0.2	33	0.2	12	0.2	102	0.6	232	3.1
14	209	5.6	16	0.2	14	0.1	9	0.1	43	0.3	164	2.2
15	101	2.7	5	0.1	6	0.0	9	0.1	20	0.1	132	1.8
16	58	1.5	1	0.0	5	0.0	5	0.1	3	0.0	55	0.7
17	18	0.5	0	0.0	1	0.0	2	0.0	0	-	26	0.3
18	7	0.2	2	0.0	0	-	0	-	0	-	17	0.2
19	1	0.0	0	-	0	-	0	-	0	-	5	0.1

Table S6. Frequency of mortality-level thermal stress events, 1985-2012. These data correspond to histograms presented in Figure S2. Mortality-level stress defined as DHW ≥ 8 °C-weeks. Light-grey shade denotes pixels that experienced 0-2 events (<1/decade) on average, and dark-grey shade denotes pixels that experienced ≥6 events (>2/decade) on average.

**Number of events**	**Global**		**Middle East**		**Indian Ocean**		**Southeast Asia**		**Australia**		**Pacific Ocean**		**Atlantic Ocean**	
	Pixels	%	Pixels	%	Pixels	%	Pixels	%	Pixels	%	Pixels	%	Pixels	%
Average±SD	*1.3±1.8*		*3.6±2.7*		*1.0±1.2*		*0.9±1.1*		*0.7±1.4*		*1.3±1.6*		*2.4±2.5*	
0	25809	42.5	591	15.8	3200	41.9	8519	47.2	5011	64.2	6683	41.7	1805	24.3
1	15768	26.0	407	10.9	2733	35.8	5347	29.6	1557	19.9	4054	25.3	1670	22.5
2	8332	13.7	450	12.0	959	12.6	2762	15.3	627	8.0	2428	15.2	1106	14.9
3	4386	7.2	461	12.3	437	5.7	956	5.3	249	3.2	1421	8.9	862	11.6
4	2399	4.0	499	13.3	177	2.3	289	1.6	118	1.5	670	4.2	646	8.7
5	1550	2.6	469	12.5	68	0.9	111	0.6	84	1.1	380	2.4	438	5.9
6	974	1.6	326	8.7	23	0.3	52	0.3	76	1.0	179	1.1	318	4.3
7	621	1.0	212	5.7	8	0.1	14	0.1	52	0.7	120	0.7	215	2.9
8	368	0.6	138	3.7	6	0.1	3	0.0	14	0.2	52	0.3	155	2.1
9	258	0.4	106	2.8	14	0.2	8	0.0	13	0.2	30	0.2	87	1.2
10	122	0.2	53	1.4	5	0.1	3	0.0	3	0.0	8	0.0	50	0.7
11	72	0.1	24	0.6	3	0.0	2	0.0	2	0.0	1	0.0	40	0.5
12	26	0.0	7	0.2	0	-	0	-	1	0.0	0	-	18	0.2
13	16	0.0	4	0.1	0	-	0	-	0	-	0	-	12	0.2
14	8	0.0	0	-	0	-	0	-	0	-	0	-	8	0.1
15	1	0.0	0	-	0	-	0	-	0	-	0	-	1	0.0

Table S7a. Annual exposure to bleaching-level thermal stress for all reef locations globally and within reef regions, 1985-2012. These data correspond to the histograms presented in the left panels of Figure 6. The three years in which the greatest percentage of pixels experienced bleaching-level thermal stress events are labeled as 1, 2 and 3 and are shaded in grey.

	**Global**		**Middle East**		**Indian Ocean**		**Southeast Asia**		**Australia**		**Pacific Ocean**		**Atlantic Ocean**	
**Year**	Pixels	%	Pixels	%	Pixels	%	Pixels	%	Pixels	%	Pixels	%	Pixels	%
1985	1962	3.2	0	-	39	0.5	514	2.8	582	7.5	786	4.9	41	0.6
1986	4229	7.0	339	9.0	94	1.2	648	3.6	991	12.7	2142	13.4	15	0.2
1987	7068	11.6	275	7.3	1860	24.4	1333	7.4	1507	19.3	1308	8.2	785	10.6
1988	7732	12.7	887	23.7	1372	18.0	2053	11.4	1465	18.8	1740	10.9	215	2.9
1989	4017	6.6	71	1.9	43	0.6	797	4.4	1685	21.6	1320	8.2	101	1.4
1990	5154	8.5	86	2.3	323	4.2	1588	8.8	899	11.5	1275	8.0	983	13.2
1991	3306	5.4	0	-	710	9.3	537	3.0	178	2.3	1609	10.0	272	3.7
1992	2051	3.4	61	1.6	242	3.2	739	4.1	551	7.1	428	2.7	30	0.4
1993	1234	2.0	321	8.6	61	0.8	357	2.0	20	0.3	197	1.2	278	3.7
1994	3490	5.7	1414	37.7	27	0.4	1062	5.9	151	1.9	706	4.4	130	1.7
1995	10836	17.8	2065	55.1	1032	13.5	1646	9.1	993	12.7	1925	12.0	3175	42.7
1996	9630	15.9	1072	28.6	528	6.9	1932	10.7	704	9.0	5123	32.0	271	3.6
1997	5228	8.6	1007	26.9	268	3.5	549	3.0	363	4.6	1801	11.2	1240	16.7
1998	29031	47.8(1)	3151	84.1(1)	5358	70.2(1)	10238	56.7(2)	2407	30.8(1)	3569	22.3	4308	58.0(3)
1999	12491	20.6	2189	58.4	335	4.4	2204	12.2	1267	16.2	3756	23.4	2740	36.9
2000	9535	15.7	656	17.5	83	1.1	2450	13.6	513	6.6	5348	33.4(2)	485	6.5
2001	11592	19.1	1925	51.4	299	3.9	3510	19.4	259	3.3	4800	30.0	799	10.8
2002	13069	21.5	2136	57.0	554	7.3	2320	12.8	2062	26.4	4038	25.2	1959	26.4
2003	17420	28.7	2117	56.5	2767	36.3(3)	4691	26.0	1344	17.2	3530	22.0	2971	40.0
2004	14013	23.1	453	12.1	1223	16.0	3178	17.6	2105	27.0(3)	4255	26.6	2799	37.7
2005	19205	31.6(3)	1214	32.4	2668	35.0	3623	20.1	1844	23.6	4101	25.6	5755	77.4(1)
2006	12792	21.1	1545	41.2	759	9.9	1458	8.1	1365	17.5	4536	28.3	3129	42.1
2007	12225	20.1	2325	62.0	527	6.9	4189	23.2	267	3.4	2018	12.6	2899	39.0
2008	8999	14.8	1458	38.9	59	0.8	885	4.9	679	8.7	3534	22.1	2384	32.1
2009	17217	28.4	1707	45.6	1389	18.2	3829	21.2	997	12.8	5668	35.4(1)	3627	48.8
2010	28952	47.7(2)	2812	75.0(2)	3706	48.6(2)	10512	58.2(1)	2376	30.4(2)	4538	28.3	5008	67.4(2)
2011	15263	25.1	786	21.0	463	6.1	4753	26.3(3)	751	9.6	4958	30.9(3)	3552	47.8
2012	13602	22.4	2339	62.4(3)	714	9.4	3789	21.0	1088	13.9	3698	23.1	1974	26.6

Table S7b. Exposure to bleaching-level thermal stress during 7-year periods for all reef locations globally and within reef regions, historically (1985-2012) and projected (centred on 2030 and 2050). Average ± SD values correspond to the histograms presented in the right panels of Figure 6. Historical values are calculated from Table S8a. Projected values are based on CMIP5 thermal stress projections using RCP8.5.

**7-year period**	**Global**		**Middle East**		**Indian Ocean**		**Southeast Asia**		**Australia**		**Pacific Ocean**		**Atlantic Ocean**	
	Pixels	%	Pixels	%	Pixels	%	Pixels	%	Pixels	%	Pixels	%	Pixels	%
Historical	60710 pixels		3747 pixels		7633 pixels		18066 pixels		7807 pixels		16026 pixels		7431 pixels	
1985	4781	7.9	237	6.3	634	8.3	1067	5.9	1044	13.4	1454	9.1	345	4.6
-1991	±2045	±3.4	±315	±8.4	±724	±9.5	±599	±3.3	±546	±7.0	±428	±2.7	±384	±5.2
1992	8786	14.5	1299	34.7	1074	14.1	2360	13.1	741	9.5	1964	12.3	1347	18.1
-1998	±9643	±15.9	±1053	±28.1	±1920	±25.2	±3520	±19.5	±805	±10.3	±1810	±11.3	±1714	±23.1
1999	13904	22.9	1527	40.8	1133	14.8	3139	17.4	1342	17.2	4261	26.6	2501	33.7
-2005	±3357	±5.5	±745	±19.9	±1140	±14.9	±895	±5.0	±732	±9.4	±624	±3.9	±1742	±23.4
2006	15579	25.7	1853	49.5	1088	14.3	4202	23.3	1075	13.8	4136	25.8	3225	43.4
-2012	±6427	±10.6	±682	±18.2	±1222	±16.0	±3135	±17.4	±671	±8.6	±1182	±7.4	±986	±13.3
Projected	1687 pixels		114 pixels		211 pixels		454 pixels		144 pixels		553 pixels		211 pixels	
2027	1145	67.9	80	70.6	172	81.5	408	89.9	96	66.4	339	61.4	49	23.3
-2033	±221	±13.1	±34	±29.8	±15	±7.1	±43	±9.4	±35	±24.5	±109	±19.8	±14	±6.6
2047	1665	98.7	114	100.0	211	100.0	454	100.0	144	100.0	549	99.1	193	91.6
-2053	±11	±0.6	±0		±0		±0		±0		±3	±0.1	±8	±4.0

Table S8. Regional data distribution for warm-season temperature variability expressed as a percentage of the climatological range, 1985-2012. Light grey shading denotes the approximately one-quarter of sites with highest variability (>20%). These data correspond to the histograms presented in Figure S5.

**Warm-season variability**	**Middle East**		**Indian Ocean**		**Southeast Asia**		**Australia**		**Pacific Ocean**		**Atlantic Ocean**	
	Pixels	%	Pixels	%	Pixels	%	Pixels	%	Pixels	%	Pixels	%
Average±SD	*7.9±1.8*		*17.1±6.6*		*17.5±7.4*		*10.1±2.5*		*22.1±15.6*		*10.7±5.4*	
0-5	163	4.4	2	0	164	0.9	1	0	7	0	410	5.5
5-10	3069	81.9	1071	14	1420	7.9	4355	55.8	994	6.2	3631	48.9
10-15	512	13.7	2452	32.1	6258	34.6	3080	39.5	5335	33.3	1925	25.9
15-20	3	0.1	1392	18.2	5731	31.7	282	3.6	3552	22.2	978	13.2
20-25	0	-	1645	21.6	2221	12.3	87	1.1	1975	12.3	307	4.1
25-30	0	-	873	11.4	1008	5.6	2	0.0	1257	7.8	145	2
30-35	0	-	188	2.5	543	3	0	-	795	5	27	0.4
35-40	0	-	9	0.1	372	2.1	0	-	473	3	8	0.1
40-45	0	-	1	0.0	212	1.2	0	-	396	2.5	0	-
45-50	0	-	0	-	82	0.5	0	-	310	1.9	0	-
50-55	0	-	0	-	44	0.2	0	-	252	1.6	0	-
55-60	0	-	0	-	9	0.0	0	-	143	0.9	0	-
60-65	0	-	0	-	2	0.0	0	-	94	0.6	0	-
65-70	0	-	0	-	0	-	0	-	63	0.4	0	-
70+	0	-	0	-	0	-	0	-	380	2.4	0	-

Table S9. Distribution of residuals from the regression of the change in bleaching stress occurrence with warm-season temperature trend. The line separating the second and third rows corresponds to the regression line in Figure 7; and the first and fourth rows indicate residuals with magnitude exceeding one standard error of estimate (SE = 1.37). The change in bleaching stress occurrence was determined as the difference in number of bleaching-level stress events (DHW ≥ 4 °C-weeks) between the 1985-1991 and 2006-2012 periods.

**Residual**	**Pixels**	**%**
< -SE	8704	14.3
< 0 and > -SE	19363	31.9
>0 and < SE	23227	38.3
> SE	9416	15.5

Table S10. Regional data distribution for climatologically warmest month, 1985-2012. These data correspond to histograms presented in Figure S3.

Warmest month	Middle East		Indian Ocean		Southeast Asia		Australia		Pacific Ocean		Atlantic Ocean	
	Pixels	%	Pixels	%	Pixels	%	Pixels	%	Pixels	%	Pixels	%
January	0	-	44	0.6	296	1.6	2447	31.3	2180	13.6%	0	-
February	0	-	419	5.5	158	0.9	3835	49.1	3897	24.3%	2	0.0
March	0	-	1249	16.4	290	1.6	338	4.3	3384	21.1%	97	1.3
April	0	-	4276	56.0	3222	17.8	641	8.2	443	2.8%	194	2.6
May	72	1.9	920	12.1	4964	27.5	22	0.3	338	2.1%	72	1.0
June	68	1.8	564	7.4	5351	29.6	0	-	438	2.7%	87	1.2
July	33	0.9	6	0.1	651	3.6	0	-	58	0.4%	44	0.6
August	2040	54.4	0	-	769	4.3	0	-	80	0.5%	1586	21.3
September	573	15.3	0	-	114	0.6	0	-	959	6.0%	3573	48.1
October	934	24.9	151	2.0	410	2.3	0	-	1309	8.2%	1738	23.4
November	27	0.7	1	0.0	968	5.4	29	0.4	1586	9.9%	38	0.5
December	0	-	3	0.0	873	4.8	495	6.3	1354	8.4%	0	-

Table S11. Regional data distribution for mean onset (month) of bleaching-level thermal stress events, 1985-2012. These data correspond to histograms presented in Figure S4.

Mean onset	Middle East		Indian Ocean		Southeast Asia		Australia		Pacific Ocean		Atlantic Ocean	
	Pixels	%	Pixels	%	Pixels	%	Pixels	%	Pixels	%	Pixels	%
January	0	-	696	9.6	1555	9.2	3859	57.0	3773	25.8	6	0.1
February	0	-	1431	19.8	762	4.5	1573	23.2	3344	22.8	81	1.1
March	0	-	1437	19.9	378	2.2	176	2.6	846	5.8	144	2.0
April	12	0.3	2915	40.4	929	5.5	15	0.2	78	0.5	34	0.5
May	55	1.5	432	6.0	2962	17.6	0	-	45	0.3	7	0.1
June	83	2.2	36	0.5	2830	16.8	0	-	144	1.0	143	2.0
July	778	20.8	48	0.7	2574	15.3	0	-	571	3.9	2569	35.3
August	2324	62.2	59	0.8	983	5.8	0	-	1493	10.2	3314	45.5
September	464	12.4	45	0.6	472	2.8	0	-	1240	8.5	975	13.4
October	21	0.6	39	0.5	413	2.4	0	-	469	3.2	6	0.1
November	1	0.0	16	0.2	1043	6.2	92	1.4	503	3.4	0	-
December	1	0.0	66	0.9	1973	11.7	1057	15.6	2129	14.5	2	0.0
No Events	8	0.2	413	5.7	1192	7.1	1035	15.3	1391	9.5	150	2.1

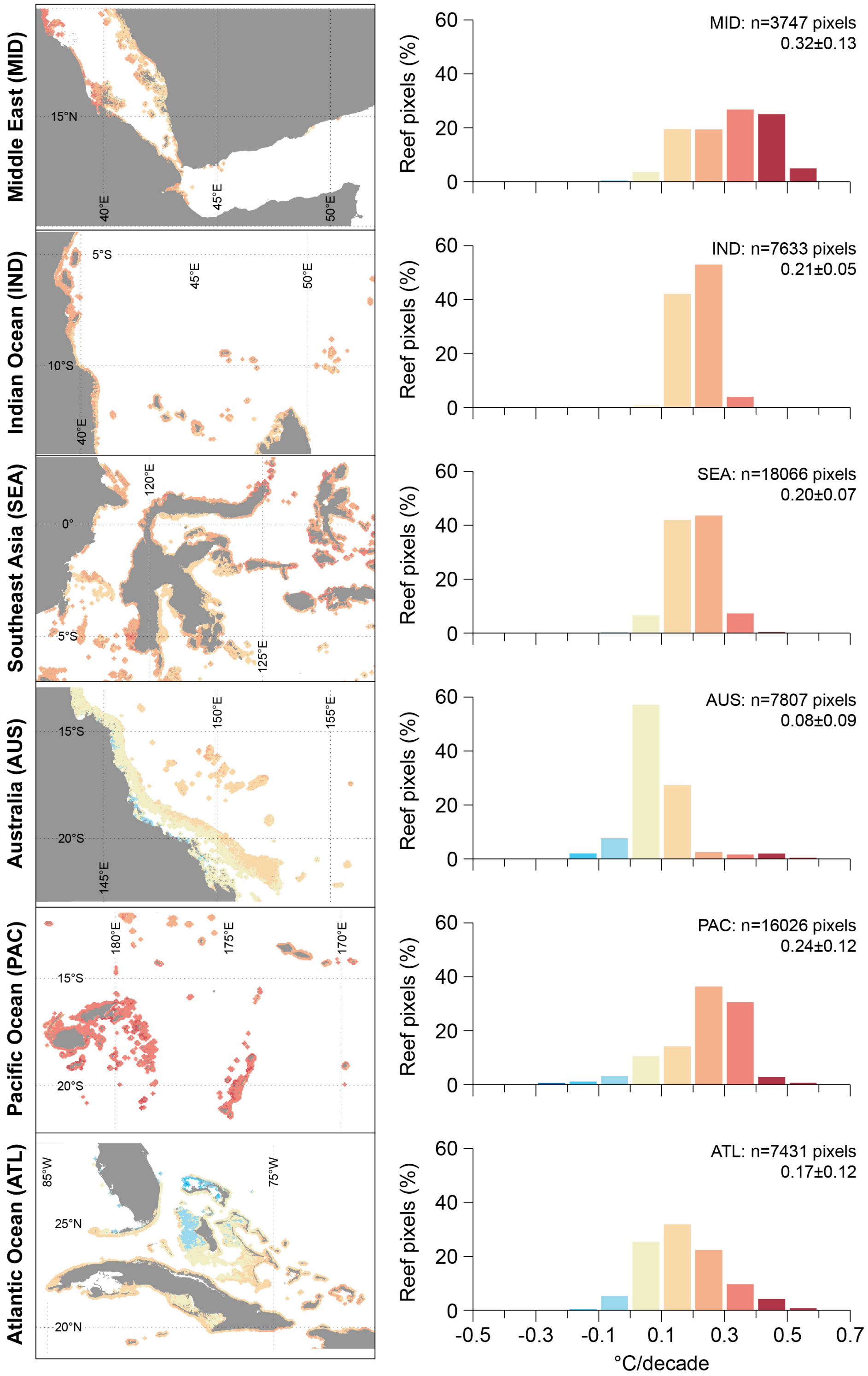

Fig. S1. **Trend in annual temperatures among reef regions, 1985-2012.** Trend values are in °C/decade. Maps (left) show results for a subset of each region; histograms (right) show the distribution of results in the full region with the regional average ± one standard deviation. Reef regions are as per Burke et al. 2011. Data are provided for each histogram in Table S2. Data visualisations produced using IDL [8.3] (Exelis Visual Information Solutions, Boulder, Colorado).

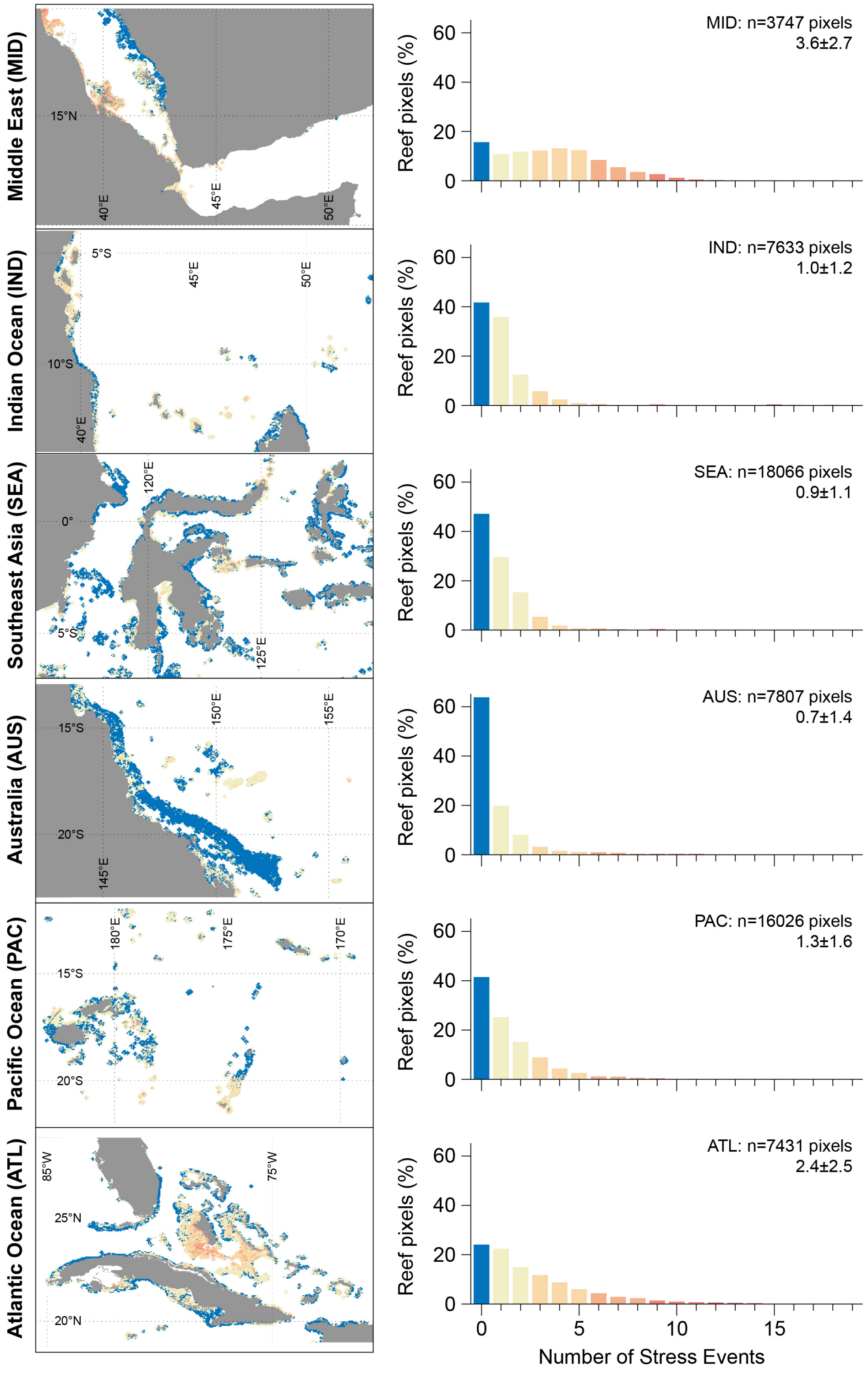

Fig. S2 **Frequency of mortality-level thermal stress events among reef regions, 1985-2012.** Mortality-level stress defined as DHW ≥ 4 °C-weeks. Maps (left) show results for a subset of each region; histograms (right) show the distribution of results in the full region with the regional average ± one standard deviation. Reef regions are as per Burke et al. 2011. Data are provided for each histogram in Table S6. Data visualisations produced using IDL [8.3] (Exelis Visual Information Solutions, Boulder, Colorado).

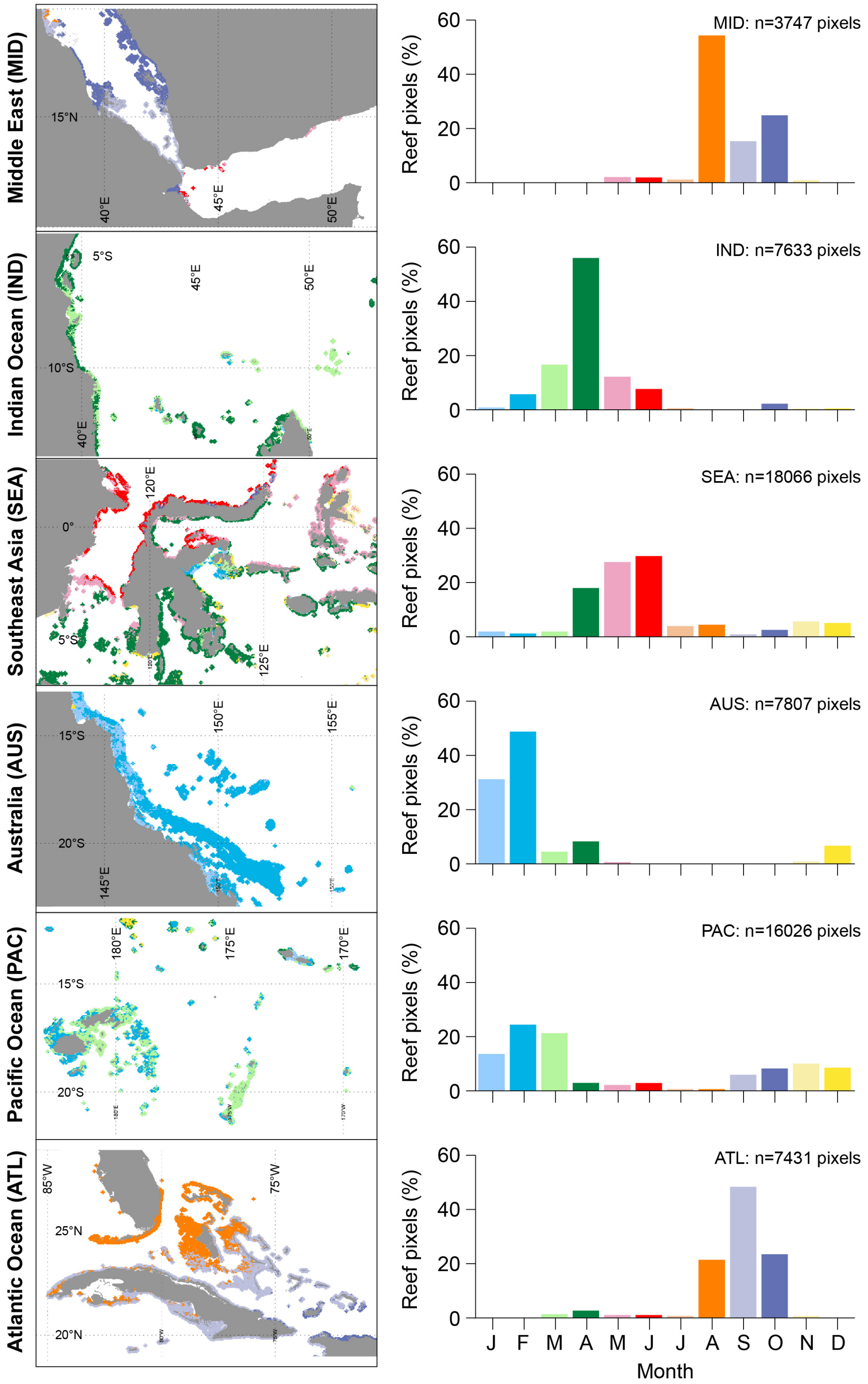

Fig. S3. **Distribution of climatologically Warmest Month among reef regions, 1985-2012.** Maps (left) show results for a subset of each region; histograms (right) show the distribution of results in the full region. Reef regions are as per Burke et al. 2011. Data are provided for each histogram in Table S10. Data visualisations produced using IDL [8.3] (Exelis Visual Information Solutions, Boulder, Colorado).

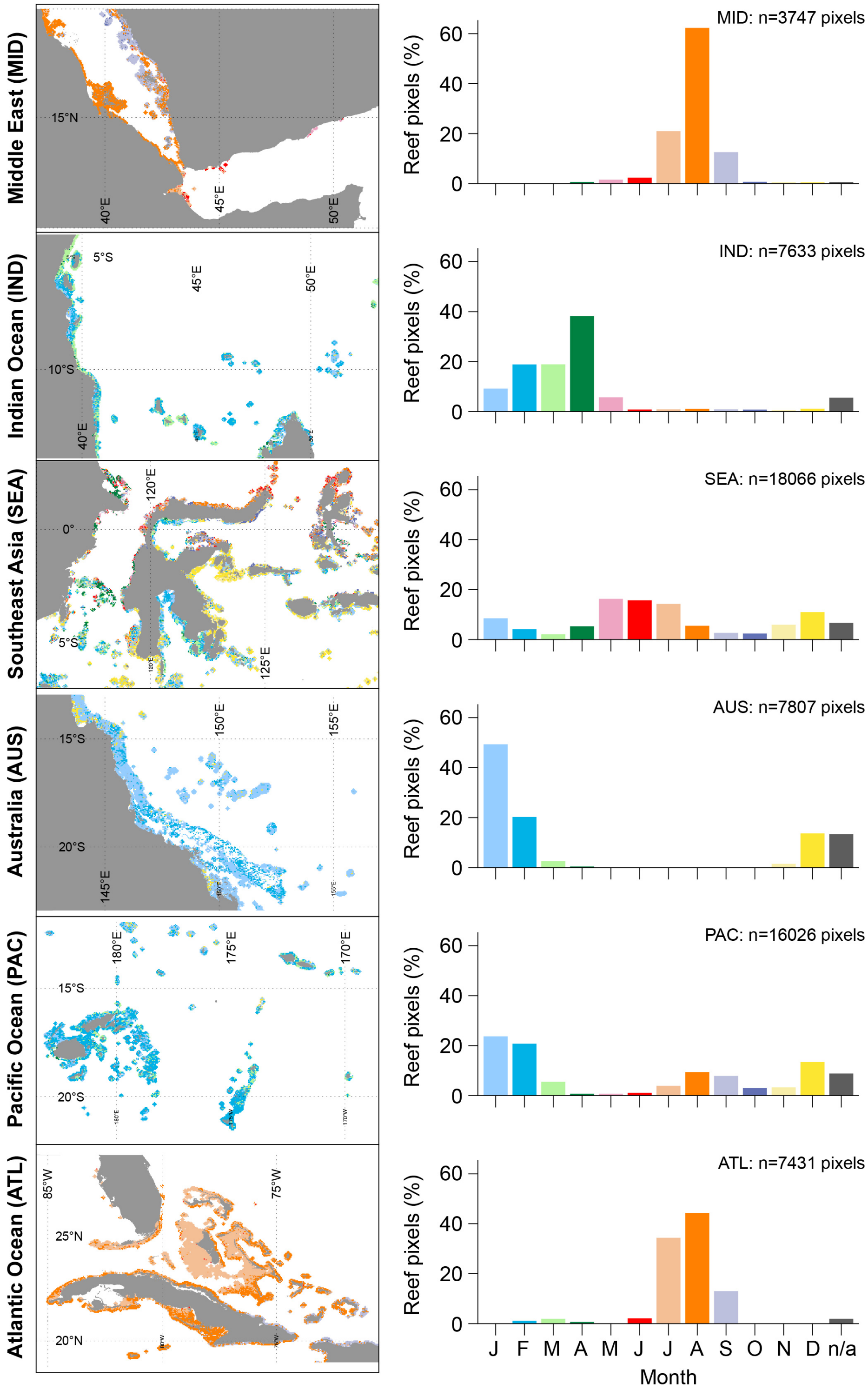

Fig. S4. **Distribution of Mean Onset (month) among reef regions, 1985-2012.** Maps (left) show results for a subset of each region; histograms (right) show the distribution of results in the full region. Reef regions are as per Burke et al. 2011. Data are provided for each histogram in Table S11. Data visualisations produced using IDL [8.3] (Exelis Visual Information Solutions, Boulder, Colorado).

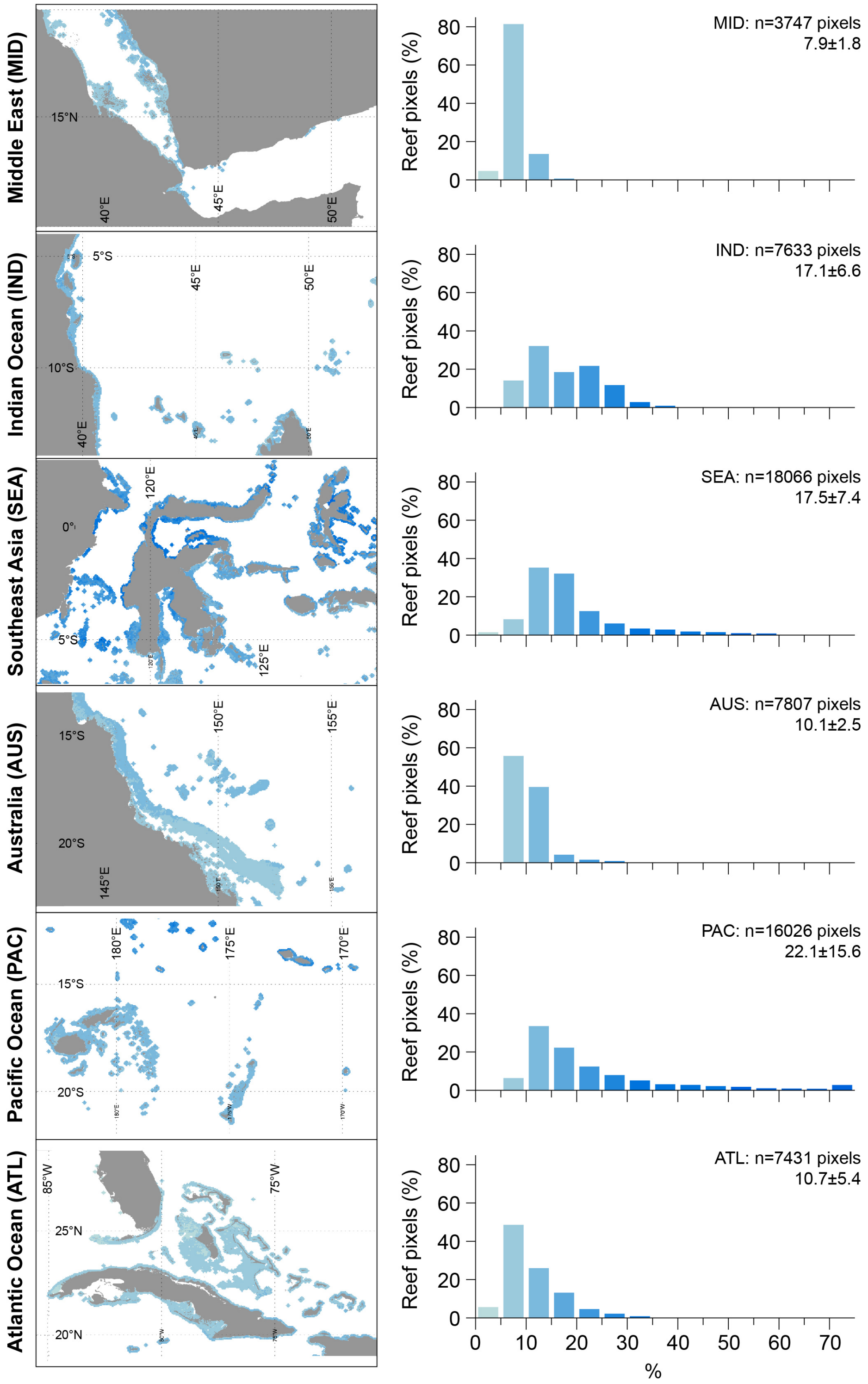

Fig. S5. **Warm-season variability among reef regions, 1985-2012.** Values are expressed as a percentage of the climatological range. Maps (left) show results for a subset of each region; histograms (right) show the distribution of results in the full region with the regional average ± one standard deviation. Reef regions are as per Burke et al. 2011. Data are provided for each histogram in Table S8. Data visualisations produced using IDL [8.3] (Exelis Visual Information Solutions, Boulder, Colorado).

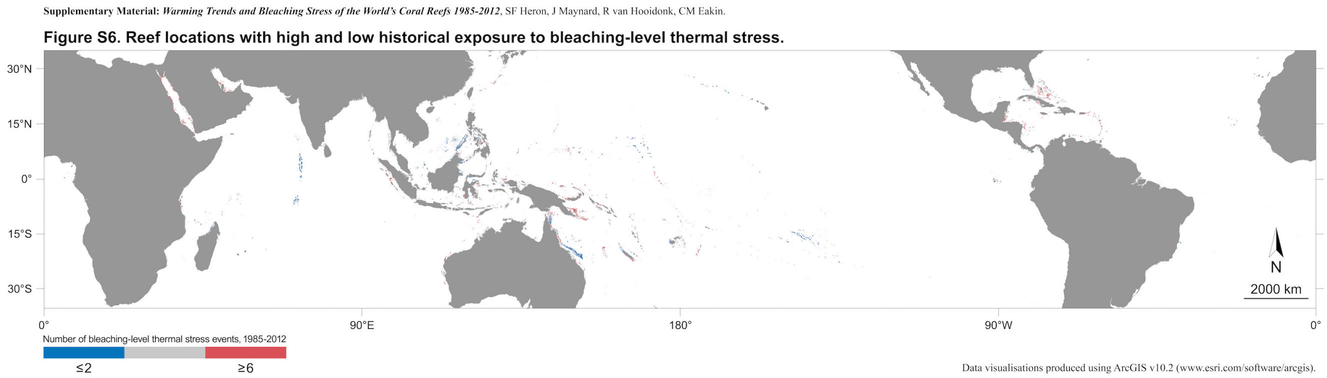

Fig. S6. **Reef locations with high and low historical exposure to bleaching-level thermal stress.** High-resolution map (available from coralreefwatch.noaa.gov/publications/HeronEtAl_2016.php) identifies reefs for which the frequency of thermal stress events during 1985-2012 that reached or exceeded Degree Heating Weeks of 4 °C-weeks was less than 1/decade (low) or was greater than 2/decade (high). Data visualisations produced using ArcGIS v10.2 (www.esri.com/software/arcgis).

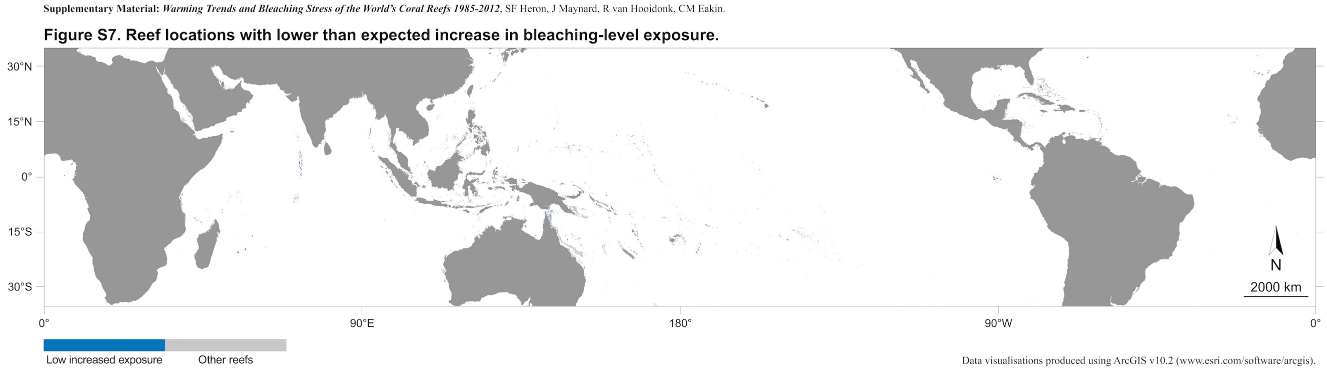

Fig. S7. **Reef locations with lower than expected increase in bleaching-level exposure.** High-resolution map (available from coralreefwatch.noaa.gov/publications/HeronEtAl_2016.php) identifies reefs for which the difference in number of bleaching-level stress events (DHW ≥ 4 °C-weeks) between the 1985-1991 and 2006-2012 periods was less than expected based on the local warm-season trend. The relationship between these is shown in Figure 7. Data visualisations produced using ArcGIS v10.2 (www.esri.com/software/arcgis).

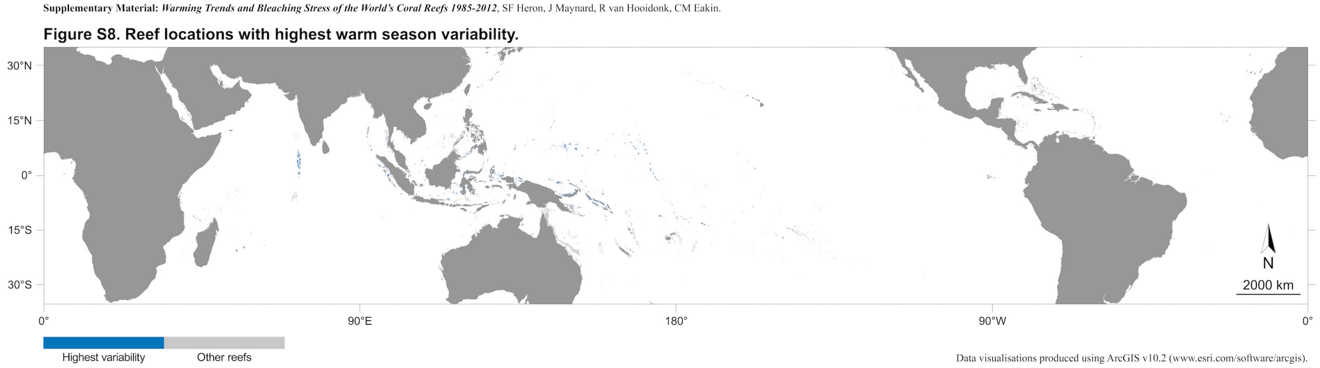

Fig. S8. **Reef locations with highest warm-season variability.** High-resolution map (available from coralreefwatch.noaa.gov/publications/HeronEtAl_2016.php) identifies reefs for which the variability in warm-season temperature was at least 20% of the climatological range in temperature. Data visualisations produced using ArcGIS v10.2 (www.esri.com/software/arcgis).
